# Efficient edit distance with duplications and contractions

**DOI:** 10.1186/1748-7188-8-27

**Published:** 2013-10-29

**Authors:** Tamar Pinhas, Shay Zakov, Dekel Tsur, Michal Ziv-Ukelson

**Affiliations:** 1 Department of Computer Science, Ben-Gurion University of the Negev, Be’er Sheva, Israel; 2 Department of Computer Science and Engineering, University of California at San Diego, La Jolla, CA, USA

**Keywords:** Edit distance, Minisatellites, Min-plus matrix multiplication, Four Russians

## Abstract

We propose three algorithms for string edit distance with duplications and contractions. These include an efficient general algorithm and two improvements which apply under certain constraints on the cost function. The new algorithms solve a more general problem variant and obtain better time complexities with respect to previous algorithms. Our general algorithm is based on min-plus multiplication of square matrices and has time and space complexities of *O *(|*Σ*|*MP *(*n*)) and *O *(|*Σ*|*n*^2^), respectively, where |*Σ*| is the alphabet size, *n* is the length of the strings, and *MP *(*n*) is the time bound for the computation of min-plus matrix multiplication of two *n* × *n* matrices (currently,

$\text{MP}(n)=O\left(\frac{n^{3}\overset{3}{\text{log}}\text{log}n}{\overset{2}{\text{log}}n}\right)$ due to an algorithm by Chan).

For integer cost functions, the running time is further improved to $O\left(\frac{|\Sigma |n^{3}}{\overset{2}{\text{log}}n}\right)$. In addition, this variant of the algorithm is online, in the sense that the input strings may be given letter by letter, and its time complexity bounds the processing time of the first *n* given letters. This acceleration is based on our efficient matrix-vector min-plus multiplication algorithm, intended for matrices and vectors for which differences between adjacent entries are from a finite integer interval *D*. Choosing a constant $\frac{1}{\underset{\left|D\right|}{\text{log}}n}<\lambda <1$, the algorithm preprocesses an *n* × *n* matrix in $O\left(\frac{n^{2+\lambda }}{\left|D\right|}\right)$ time and $O\left(\frac{n^{2+\lambda }}{|D|\lambda^{2}\overset{2}{\underset{\left|D\right|}{\text{log}}}n}\right)$ space. Then, it may multiply the matrix with any given *n*-length vector in $O\left(\frac{n^{2}}{\lambda^{2}\overset{2}{\underset{\left|D\right|}{\text{log}}}n}\right)$ time. Under some discreteness assumptions, this matrix-vector min-plus multiplication algorithm applies to several problems from the domains of context-free grammar parsing and RNA folding and, in particular, implies the asymptotically fastest $O\left(\frac{n^{3}}{\overset{2}{\text{log}}n}\right)$ time algorithm for single-strand RNA folding with discrete cost functions.

Finally, assuming a different constraint on the cost function, we present another version of the algorithm that exploits the run-length encoding of the strings and runs in $O\left(\frac{\left|\Sigma |\text{nMP}(ñ\right)}{ñ}\right)$ time and O(|Σ|nñ) space, where ñ is the length of the run-length encoding of the strings.

## Background

Comparing strings is a well-studied problem in computer science as well as in bioinformatics. Traditionally, string similarity is measured in terms of *edit distance*, which reflects the minimum-cost edit of one string to the other, based on the edit operations of substitutions (including matches) and deletions/insertions (indels). In this paper, we address the problem of string edit distance with the additional operations of duplication and contraction. The motivation for this problem originated from the study of minisatellites and their comparisons in the context of population genetics [1].

### Motivation: minisatellite comparison

A minisatellite is a section of DNA that consists of tandem repetitions of short (6–100 nucleotides) sequence motifs spanning 500 nucleotides to several thousand nucleotides. The repeated motifs also vary in sequence through base substitutions and indels. For one minisatellite locus, both the type and the number of motifs vary between individuals in a population. Therefore, pairwise comparisons of minisatellites are typically applied in studying the evolution of populations.

A *minisatellite map* represents a minisatellite region, where each motif is encoded by a letter and handled as one entity (denoted *unit*). When comparing minisatellite maps, one has to consider that regions of the map have arisen as a result of duplication events from the neighboring units. The single copy duplication model, where only one unit can duplicate at a time, is the most popular and its biological validation was asserted for the MSY1 minisatellites [1,2]. According to this model, one unit can mutate to another unit via an indel or a mutation of a single nucleotide within it. Also, a unit can be duplicated, that is, an additional copy of the unit may appear next to the original one in the map (tandem repeat). Thus, when comparing minisatellite maps, two additional operations are considered: unit duplication and unit contraction.

### The EDDC problem definition

The single copy duplication model of minisatellite maps gave rise to a new variant of the string edit distance problem, *Edit Distance with Duplications and Contractions* (EDDC), which allows five edit operations: insertion, deletion, mutation, duplication and contraction.

We start with some string notations. Let *s* be a string. Denote by *s*_*i*_ the *i*-th letter in *s*, starting at index 0, and by *s*_*i*,*j*_ the substring *s*_*i*_*s*_*i*+ 1_ … *s*_*j*- 1_ of *s*. A substring of the form *s*_*i*,*i*_ is an empty string, which will be denoted by *ε*. We use superscripts to denote substrings without an explicit indication of their start and end positions, and write e.g. *s *= *s*^*a *^*s*^*b *^ to indicate that *s* is a concatenation of the two substrings *s*^*a*^ and *s*^*b*^.

In the edit distance problem, one is given a source string *s* and a target string *t* over a finite alphabet *Σ*. An *edit script* from *s* to *t* is a series of strings $ES=\langle s=u^{0},u^{1},u^{2},\ldots ,u^{r}=t\rangle$, where each intermediate string *u*^*i*^ is obtained by applying a single edit operation to the preceding string *u*^*i*-1^. In the standard problem definition [3-5], the allowed edit operations are *insertion* of a letter at some position in an intermediate string *u*^*i*^, *deletion* of a letter in *u*^*i*^, and *mutation* of a letter in *u*^*i*^ to another letter. The single-copy EDDC problem variant adds two operations: *duplication* - inserting into *u*^*i*^ a letter in a position adjacent to a position that already contains the same letter, and *contraction* - deleting from *u*^*i*^ one copy of a letter where there are two consecutive copies of this letter. Denote by *ins *(*α*),*dup *(*α*) and *del *(*α*) costs associated with the insertion, duplication and deletion operations applied to a letter *α* in the alphabet, respectively, by *cont *(*α*) the cost of contracting two consecutive occurrences of *α* into a single occurrence, and by *mut *(*α*,*β*) the cost of mutating *α* to a letter *β*. Define the *cost* of ES to be the summation of its implied operation costs, and the *length *|ES|=r of ES to be the number of operations performed in ES. Clearly, for every pair of strings *s* and *t*, there is some script transforming *s* to *t*, e.g. the script that first deletes all letters in *s* and then inserts all letters in *t*. An optimal edit script from *s* to *t* is one which has a minimum cost. The *edit distance* from *s* to *t*, denoted by *ed *(*s*,*t*), is the cost of an optimal edit script from *s* to *t*. The goal of the EDDC problem is, given strings *s* and *t*, to compute *ed* (*s*,*t*).

Previous algorithms assume various constraints on operation costs (see Section “A comparison with previous works”). In this paper, the only limiting assumption made is that all operation costs are nonnegative. In addition, we can make the following assumption without loss of generality, which will be required by the algorithms presented in this paper:

#### 

**Property 1.** *It may be assumed without loss of generality that for every α*,*β* ∈ *Σ*, 

• *ins *(*α*) = *ed *(*ε*,*α*), *del *(*α*) = *ed *(*α*,*ε*),

• *dup *(*α*) = *ed *(*α*,*α α*), *cont *(*α*) = *ed *(*α α*,*α*),

• *mut *(*α*,*β*) = *ed *(*α*,*β*).

This assumption can be made, since in case one of the operation costs violates the assumption, then such an operation can always be replaced by a series of operations that would induce the same modification at lower cost. For example, it cannot be that *mut *(*α*,*β*) < *ed *(*α*,*β*), since *ed *(*α*,*β*) is smaller then or equal to the cost of any script from *α * to *β*, among which is the script containing the single mutation operation of *α* into *β*. Moreover, if *mut *(*α*,*β*) > *ed *(*α*,*β*), then we can always replace any mutation of *α * into *β* by a series of operations that transform *α* into *β* at cost *ed *(*α*,*β*). In this case, we may simply assume that *mut *(*α*,*β*) = *ed *(*α*,*β*), and interpret any such a mutation appearing in a script as being implem ented by the corresponding series of operations. In particular, Property 1 implies that *mut *(*α*,*α*) = 0 (since all operation costs are nonnegative, *ed *(*w*,*w*) = 0 for every string *w*), *dup *(*α*) ≤ *ins *(*α*) (since the cost of the script from *α* to *α α* that applies a single insertion of *α* is *ins *(*α*) ≥ *ed *(*α*,*α α*) = *dup *(*α*)), *cont *(*α*) ≤ *del *(*α*), and *mut *(*α*,*β*) ≤ *mut *(*α*,*γ*) + *mut *(*γ*,*β*) for every *γ* ∈ *Σ*.

Insertions and duplications are considered to be *generating* operations, increasing by one letter the length of the string. Similarly, deletions and contractions are considered to be *reducing* operations, decreasing by one letter the length of the string. An edit script containing no reducing operation is called a *non-reducing* script, and an edit script containing no generating operation is called a *non-generating* script.

### Previous work

The EDDC problem was first defined by Bérard and Rivals [2], who suggested an *O *(*n*^4^) time and *O *(*n*^3^) space algorithm for the problem, where *n* is the length of the two input strings (for the sake of simplicity, we assume that both strings are of the same length). This was followed by the work of Behzadi and Steyaert [6], who gave an *O *(|*Σ*|*n*^3^) time and *O *(|*Σ*|*n*^2^) space algorithm for the problem, where |*Σ*| is the alphabet size (typically a few tens of unique units). Behzadi and Steyaert [7] improved their algorithms’ complexity, based on run-length encoding, to $O(n^{2}+n{ñ}^{2}+|\Sigma |{ñ}^{3}+|\Sigma {|}^{2}{ñ}^{2})$ time and $O\left(|\Sigma |(n+{ñ}^{2})+n^{2}\right)$ space, where ñ is the length of the run-length encoding of the input strings. Run-length encoding was also used by Bérard et al. [8], who proposed an $O(n^{3}+|\Sigma |{ñ}^{3})$ time and $O(n^{2}+|\Sigma |{ñ}^{2})$ space algorithm. Abouelhoda et al. [9] gave an algorithm with an alphabet size independent time and space complexities of $O(n^{2}+n{ñ}^{2})$ and *O*(*n*^2^), respectively. A detailed comparison between the different problem models appears in Section “A comparison with previous works”.

### Our contribution

This paper presents several algorithms for EDDC which are currently the most general and efficient for the problem. 

1. We give an algorithm for EDDC for general non-negative cost functions that is based on min-plus square matrix multiplication. This algorithm is an adaptation of the framework of [10] (see also [11]). For two input strings over an alphabet *Σ* and of length *n* each, the time and space complexities of this algorithm are *O *(|*Σ*|*MP *(*n*)) and *O *(|*Σ*|*n*^2^), respectively, where *MP *(*n*) is the time complexity of a min-plus multiplication of two *n* × *n* matrices. Using the matrix multiplication algorithm of Chan [12], this algorithm runs in $O\left(\frac{|\Sigma |n^{3}\overset{3}{\text{log}}\text{log}n}{\overset{2}{\text{log}}n}\right)$ time (Section “A matrix multiplication based algorithm for EDDC”). Moreover, our algorithm applies less restrictions on the cost function with respect to previous algorithms and is currently the only algorithm that works for the most general problem settings (Section “A comparison with previous works”).

2. We describe a more efficient algorithm for EDDC when all operation costs are integers. This algorithm can also be applied in an online setting, where in each step a letter is added to one of the input strings. The time complexity of processing *n* letters in the input is $O\left(\frac{|\Sigma |n^{3}}{\overset{2}{\text{log}}n}\right)$, where the base of the log function is determined by the range of cost values (Section “An online algorithm for EDDC using min-plus matrix-vector multiplication for discrete cost functions”). In order to achieve this, we obtained the following stepping-stone results, which are of interest on their own. 

(a) Let *A* be an *n* × *m* matrix for which differences between adjacent entries are within some finite integer interval *D*. Choosing a time/space complexity tradeoff parameter *λ*, where $\frac{1}{\underset{\left|D\right|}{\text{log}}(n+m)}<\lambda <1$, we describe a preprocessing algorithm for *A* that runs in $O\left(\frac{\text{nm}{\left(n+m\right)}^{\lambda }}{\left|D\right|}\right)$ time and requires $O\left(\frac{\text{nm}{\left(n+m\right)}^{\lambda }}{\left|D|\lambda^{2}\overset{2}{\underset{\left|D\right|}{\text{log}}}(n+m\right)}\right)$ space. This preprocessing allows later to compute min-plus multiplications between *A* and *m*-length vectors sustaining the same discreteness requirement in $O\left(\frac{\text{nm}}{\lambda^{2}\overset{2}{\underset{\left|D\right|}{\text{log}}}(n+m)}\right)$ time (Section “An efficient *D*-discrete min-plus matrix-vector multiplication algorithm”). The algorithm is an adaptation of Williams’ matrix-vector multiplication algorithm over a finite semiring [13], with some notions similar to those in Frid and Gusfield’s RNA folding algorithm [14].

(a) The manner in which the new matrix-vector multiplication algorithm is integrated into the EDDC algorithm can be generalized to algorithms for a family of related problems, denoted VMT problems [11], under certain discreteness assumptions. This family includes many important problems from the domains of RNA folding and CFG parsing. An example of such a problem is the *single strand RNA folding problem*[15] under discrete scoring schemes. Our new matrix-vector multiplication algorithm can be integrated into an algorithm for the latter problem to yield an $O\left(\frac{n^{3}}{\overset{2}{\text{log}}n}\right)$ time algorithm, improving the best previously known asymptotic time bound for the problem (see Section “Online VMT algorithms”).

3. We extend our approach to exploit run-length encodings of the input strings, assuming some restrictions on the cost functions. This reduces the time and space complexities of the algorithm to $O\left(|\Sigma |n^{2}+\frac{\left|\Sigma |\text{nMP}(ñ\right)}{ñ}\right)$ and $O\left(|\Sigma |\mathrm{nñ}\right)$, respectively, where ñ is the length of the run-length encoding of the input (Section “Additional acceleration using run-length encoding”).

The rest of the paper is organized as follows. In Section “A baseline algorithm for the EDDC problem”, a recursive computation for EDDC and its implementation using dynamic programming (DP) is presented. Section “A matrix multiplication based algorithm for EDDC” shows how to accelerate the algorithm by incorporating efficient min-plus matrix multiplication subroutines. In Section “An online algorithm for EDDC using min-plus matrix-vector multiplication for discrete cost functions”, an efficient min-plus matrix-vector multiplication algorithm is described for matrices and vectors which differences between adjacent entries are taken from a finite integer interval. This algorithm can be used for obtaining an accelerated online version of the EDDC algorithm, as well as for improving time complexities of several related problems. Section “Additional acceleration using run-length encoding” describes a variant of the EDDC algorithm that exploits run-length encoding. Comparison between this and previous works is given in Section “A comparison with previous works”, and Section “Conclusions and discussion” gives a concluding discussion. Additional proofs omitted from the main manuscript are given in the Appendix.

## A baseline algorithm for the EDDC problem

In this section, we give a simple algorithm for the EDDC problem. We start by showing some recursive properties of the problem, and then formulate a straightforward dynamic programming implementation for the recursive computation.

### The recurrence formula

Our recursive formulas resemble previous formulations [6,9], yet solve a slightly more general variant of the problem (see discussion in Section “A comparison with previous works”). Since the proof of correctness of these recursive formulas is similar to previous ones, we defer it to Appendix “Correctness of the recursive computation”.

A (strict) *partition* of a string *w* of length at least 2 is a pair of strings (*w*^*a*^,*w*^*b*^), such that *w* = *w*^*a *^*w*^*b *^and *w*^*a*^,*w*^*b *^≠ *ε*. Denote by *P *(*w*) the set of all partitions of *w*. For example, for *w* = *abac *, *P*(*w*) = {(*a*,*bac*),(*ab*,*ac*),(*aba*,*c*)}.

**Figure 1:**
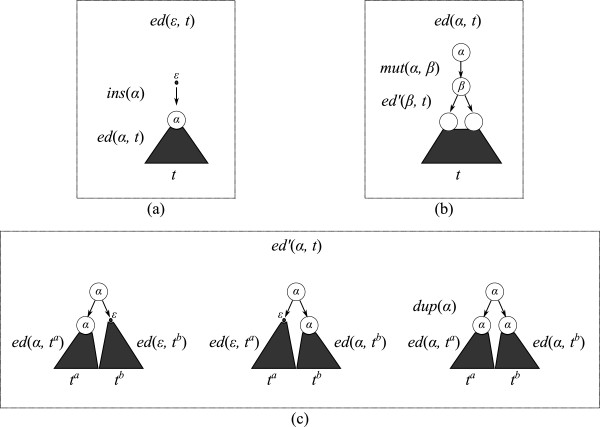
**Edit distance computation for the case where the source string is either empty or contains a single letter.** Charts **(a)**, **(b)** and **(c)** exemplify Equations 1, 2 and 3, respectively.

For a source string which is either empty or contains a single letter and a target string *t*, Equations 1 to 3 (Figure 1) describe a recursive EDDC computation. This computation interleaves, in a mutually recursive manner, the computation of an additional special value *ed*^′ ^(*α*,*t*), where *ed*^′ ^(*α*,*t*) is defined to be the minimum cost of a non-reducing edit script from *α* to *t* that does not start with a mutation (*t* is required to contain at least two letters). 

(1) $$\;\begin{array}{l} \text{ed}(\varepsilon ,t)=\left\{\begin{array}{ll} 0, & t=\varepsilon ,\\ \text{min}\left\{\text{ins}(\alpha )+\text{ed}(\alpha ,t)\;|\;\alpha \in \Sigma \right\}, & \text{otherwise}. \end{array}\right) \end{array}$$

(2) $$\;\begin{array}{l} \text{ed}(\alpha ,t)=\left\{\begin{array}{ll} \text{del}(\alpha ), & t=\varepsilon ,\\ \text{mut}(\alpha ,\beta ), & t=\beta ,\\ \text{min}\left\{\text{mut}(\alpha ,\beta )+ed^{\prime }(\beta ,t)\;|\;\beta \in \Sigma \right\}, & \text{otherwise}. \end{array}\right) \end{array}$$

(3) $$\;\begin{array}{l} ed^{\prime }(\alpha ,t)\\ \;=\text{min}\left\{\left(\begin{array}{l} \text{ed}\left(\alpha ,t^{a}\right)+\text{ed}\left(\varepsilon ,t^{b}\right),\\ \text{ed}\left(\varepsilon ,t^{a}\right)+\text{ed}\left(\alpha ,t^{b}\right),\\ \text{dup}(\alpha )+\text{ed}\left(\alpha ,t^{a}\right)+\text{ed}\left(\alpha ,t^{b}\right) \end{array}\right|\left(t^{a},t^{b}\right)\in P(t)\right\}\\ \;(t\;\text{is of length}\geq 2) \end{array}$$

Symmetrically, Equations 4 to 6 give the recursive computation for a source string *s* and a target string which is either empty or contains a single letter. Here, *ed*^′ ^(*s*,*α*) is defined as the minimum cost of a non-generating edit script from *s* to *α* which does not end with a mutation (*s* is required to contain at least two letters).

(4) $$\;\begin{array}{l} \text{ed}(s,\varepsilon )=\left\{\begin{array}{ll} 0, & s=\varepsilon ,\\ \text{min}\left\{\text{ed}(s,\alpha )+\text{del}(\alpha )\;|\;\alpha \in \Sigma \right\}, & \text{otherwise}. \end{array}\right) \end{array}$$

(5) $$\;\begin{array}{l} \text{ed}(s,\alpha )=\left\{\begin{array}{ll} \text{ins}(\alpha ), & s=\varepsilon ,\\ \text{mut}(\beta ,\alpha ), & s=\beta ,\\ \text{min}\left\{ed^{\prime }(s,\beta )+\text{mut}(\beta ,\alpha )\;|\;\beta \in \Sigma \right\}, & \text{otherwise}. \end{array}\right) \end{array}$$

(6) $$\;\begin{array}{l} ed^{\prime }(s,\alpha )\\ \;=\text{min}\left\{\left(\begin{array}{l} \text{ed}\left(s^{a},\alpha \right)+\text{ed}\left(s^{b},\varepsilon \right),\\ \text{ed}\left(s^{a},\varepsilon \right)+\text{ed}\left(s^{b},\alpha \right),\\ \text{ed}\left(s^{a},\alpha \right)+\text{ed}\left(s^{b},\alpha \right)+\text{cont}(\alpha ) \end{array}\right|\left(s^{a},s^{b}\right)\in P(s)\right\}\\ \;(s\;\text{is of length}\geq 2) \end{array}$$

**Figure 2:**
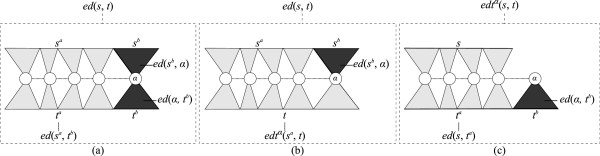
**Edit distance computation when both input strings are of length at least 2.** Chart **(a)** illustrates Equation 7, while charts **(b)** and **(c)** illustrate its decomposition into Equations 8 and 9, respectively.

In case both source string *s* and target string *t* are of length at least 2, the following equation can be used for computing *ed *(*s*,*t*) (Figure 2(a)): 

(7) $$\;\begin{array}{l} \text{ed}(s,t)\\ \;=\text{min}\left\{\begin{array}{l} \text{ed}(s,\alpha )+\text{ed}(\alpha ,t),\\ \text{ed}\left(s^{a},t^{a}\right)+\text{ed}\left(s^{b},\alpha \right)+\text{ed}\left(\alpha ,t^{b}\right) \end{array}\left|\begin{array}{l} \left(s^{a},\;s^{b}\right)\in P(s),\\ \left(t^{a},\;t^{b}\right)\in P(t),\\ \alpha \in \Sigma \end{array}\right)\right\}\\ \;(s\;\text{and}\;t\;\text{are of lengths}\geq 2) \end{array}$$

For allowing efficient computation, Equation 7 can be replaced by Equations 8 and 9, which are computed in a mutually recursive manner to yield an equivalent computation (Figure 2(b) and Figure 2(c), respectively).

(8) $$\;\begin{array}{ll} \text{ed}(s,t)= & \text{min}\left\{\begin{array}{l} \text{ed}(s,\alpha )+\text{ed}(\alpha ,t),\\ \text{ed}t^{\alpha }\left(s^{a},t\right)+\text{ed}\left(s^{b},\alpha \right) \end{array}\left|\begin{array}{l} \left(s^{a},\;s^{b}\right)\in P(s)\\ \alpha \in \Sigma \end{array}\right)\right\}\\ & \left(s\;\text{and}\;t\;\text{are of lengths}\geq 2\right) \end{array}$$

(9) $$\;\begin{array}{ll} \text{ed}t^{\alpha }(s,t)= & \text{min}\left\{\begin{array}{l} \text{ed}\left(s,t^{a}\right)+\text{ed}\left(\alpha ,t^{b}\right) \end{array}\left|\begin{array}{l} \left(t^{a},t^{b}\right)\in P(t) \end{array}\right)\right\}\\ & \left(t\;\text{is of lengths}\geq 2\right) \end{array}$$

All base-cases of the above recursive equations are implied from Property 1 in a straightforward manner.

#### 

**Theorem 1.** *EDDC is correctly solved by Equations 1-9*.

The proof of Theorem 1 appears in Appendix “Correctness of the recursive computation”.

### A baseline dynamic-programming algorithm for EDDC

In this section, we describe a DP algorithm implementing the recursive EDDC computation given by Equations 1 to 9, which is the basis for improvements introduced later in this paper.

Let *s * and *t* be the input source and target strings, respectively, and for simplicity assume both strings are of length *n*. The algorithm maintains the following matrices for storing solutions to sub-instances of the input which occur along the recursive computation. All matrices are of size (*n* + 1) × (*n* + 1), with row and column indices in the range 0,1,2,…,*n*. 

• For every *α* ∈ *Σ*, the algorithm maintains matrices *S*^′ *α*^, *S*^*α*^, *T*^′*α*^, and *T*^*α*^. Entries *S*^′*α*^[ *k*,*i*], *S*^*α*^[ *k*,*i*], *T*^′*α*^[ *l*,*j*] and *T*^*α*^[ *l*,*j*] are used for storing the values *ed*^′ ^(*s*_*k*,*i*_,*α*), *ed*(*s*_*k*,*i*_,*α*), *ed*^′ ^(*α*,*t*_*l*,*j*_), and *ed *(*α*,*t*_*l*,*j*_), respectively.

• Two matrices *S*^*ε *^and *T*^*ε*^, whose entries *S*^*ε *^[ *k*,*i*] and *T*^*ε *^[ *l*,*j*] are used for storing values of the forms *ed *(*s*_*k*,*i*_,*ε*) and *ed *(*ε*,*t*_*l*,*j*_), respectively.

• For every *α* ∈ *Σ*, a matrix *EDT*^*α *^whose entries *EDT*^*α *^[ *i*,*j*] are used for storing the values *edt*^*α *^(*s*_0,*i*_,*t*_0,*j*_).

• A matrix *ED*, whose entries *ED *[ *i*,*j*] are used for storing the values *ed *(*s*_0,*i*_,*t*_0,*j*_).

The algorithm consists of two stages: Stage 1 computes solutions to all sub-instances in which one of the substrings is either empty or single-lettered, applying Equations 1 to 6. Stage 2 uses the values computed in Stage 1 in order to compute all prefix-to-prefix solutions *ed *(*s*_0,*i*_,*t*_0,*j*_) and *edt *^*α *^(*s*_0,*i*_,*t*_0,*j*_) according to Equations 8 and 9. In particular, Stage 2 computes the edit distance *ed *(*s*_0,*n*_,*t*_0,*n*_) = *ed *(*s*,*t*) between the two complete strings. The entries are traversed in an order which guarantees that upon computing each entry, all solutions to sub-instances appearing on the right-hand side of the relevant equation are already computed and contained in the corresponding entries. Algorithm 1 gives the pseudo-code for this computation.

#### **Algorithm 1 BASELINE-EDDC(*s*,** ***t*)**

#### ***Complexity analysis of Algorithm 1***

The running time of Algorithm 1 is dictated by the total time required to compute all entries in the DP matrices. Each entry is computed according to one of the recursive equations, where the number of operations in such a computation depends on the number of expressions examined on the right-hand side of the corresponding recursive equation. Note that the value of each examined expression is obtained in a constant time, by querying previously computed values stored in the matrices.

The computation of each entry in matrices *T*^*ε *^and *S*^*ε *^and in matrices of the form *T*^*α *^and *S*^*α *^takes *O *(|*Σ*|) time, due to Equations 1, 4, 2, and 5, respectively. As there are *O *(|*Σ*|) such matrices and each matrix contains *O *(*n*^2^) entries, their overall computation time is *O *(|*Σ*|^2 ^*n*^2^). The computation of entries in *T *^′*α *^and *S*^′*α *^take *O *(*n*) time, due to Equations 3 and 6, respectively. There are *O *(|*Σ*|) such matrices, each of size *O *(*n*^2^), and so the total time for computing all entries in these matrices is *O *(|*Σ*|*n*^3^). According to Equation 9, computing each entry of the form *EDT*^*α *^[ *i*,*j*] takes *O *(*n*) time, and as there are *O *(|*Σ*|*n*^2^) such entries the total time for computing all these entries is *O *(|*Σ*|*n*^3^). According to Equation 8, computing each entry of the form *ED *[ *i*,*j*] takes *O *(|*Σ*|*n*) time, and since there are *O *(*n*^2^) such entries, the total time for computing all these entries is again *O *(|*Σ*|*n*^3^). Thus, the total running time of the algorithm is *O *(|*Σ*|*n*^3^ + |*Σ*|^2 ^*n*^2^). Under the assumption that |*Σ*| = *O *(*n*), the time is *O *(|*Σ*|*n*^3^). The algorithm requires *O *(|*Σ*|*n*^2^) space for maintaining the DP matrices.

## A matrix multiplication based algorithm for EDDC

In previous work by the authors [11], Vector Multiplication Templates (VMTs) were identified as templates for computational problems sustaining certain properties, such that algorithms for solving these problems can be accelerated using efficient matrix multiplication subroutines (similarly to Valiant’s algorithm for CFG recognition [10]). Intuitively, standard algorithms for VMT problems perform computations that can be expressed in terms of vector multiplications, and these computations can be computed and combined more efficiently using efficient matrix multiplications. In this section, we show that EDDC exhibits such VMT properties, and formulate a new algorithm that incorporates matrix-matrix min-plus multiplications. This algorithm yields a better running time than that of the baseline algorithm in the previous section.

### Notations for matrices

For two integers *p*,*q* such that *p* ≤ *q*, *I*_*p*,*q*_ denotes the interval of integers *I*_*p*,*q*_ = [ *p*,*p* + 1,…,*q* - 1]. We use the notation *A*_*n* × *m*_ to imply that the matrix *A* has *n* rows and *m* columns, and say that *A* has the dimensions *n* × *m* (rows and column indices start at 0). For a subset of row indices *I* and a subset of column indices *J*, denote by *I* × *J* the *region* which contains all pairs of indices (*i*,*j*), such that *i* ∈ *I* and *j* ∈ *J*. Define *A *[ *I*,*J*] to be the submatrix of *A*, which is induced by all entries in the region *I* × *J*. When *I* contains a single row *i* or *J* contains a single column *j*, we simplify the notation and write *A *[ *i*,*J*] or *A *[ *I*,*j*], respectively.

Define the following operations on matrices. Let *tr *(·) denote the *transpose* operation for matrices. For a set of matrices $A=\left\{A^{1},A^{2},\ldots ,A^{r}\right\}$ all of the same dimensions *n* × *m*, denote by $\text{min}\left\{A\right\}$ the *entry-wise min* operation over A, whose result is a matrix *C*_*n* × *m*_, such that $C[\;i,j]=\text{min}\left\{A[\;i,j]|\;A\in A\right\}$. $\text{min}\left\{A\right\}$ can be computed in $O\left(|A|\text{nm}\right)$ time in a straightforward manner. For matrices *A*_*n* × *k*_ and *B*_*k* × *m*_, the *min-plus multiplication* of *A* and *B*, denoted *A *⊗ *B*, results in a matrix *C*_*n* × *m*_, where the entries of *C* are defined by *C *[ *i*,*j*] = min{*A *[ *i*,*h*] + *B *[ *h*,*j*]| 0 ≤ *h* < *k*}. Naively, *A* ⊗ *B* can be computed in *O *(*nkm*) operations. Denote the time complexity of a min-plus multiplication of two *n* × *n* matrices by *MP *(*n*). At present, the asymptotically fastest algorithm for min-plus square matrix multiplication is that of Chan [12], taking $O\left(\frac{n^{3}\overset{3}{\text{log}}\text{log}n}{\overset{2}{\text{log}}n}\right)$ time.

In the following observation, we point out how matrix multiplication can be computed as a composition of two parts, where each of the items (1-3) in the observation addresses a partitioning in one of the three dimensions. This will be later used by our recursive computation which is based on such partitioning.

#### 

**Figure 3:**
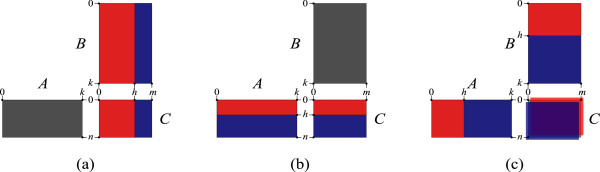
**Decomposition of a matrix-multiplication.** In all three charts, the matrix C is the result of the multiplication *A* ⊗ *B*. Charts **(a)**, **(b)**, and **(c)** illustrate items 1, 2, and 3 of Observation 1, respectively.

**Observation 1.** *Let A*_*n* × *k*_,*B*_*k* × *m *_and *C*_*n* × *m *_*be matrices, such that C* = *A* ⊗ *B* *(see Figure *3*)*. 

1. For every 0 ≤ *h* < *m*, *C *[ *I*_0,*n*_,*I*_0,*h*_] = *A* ⊗ *B *[ *I*_0,*k*_,*I*_0,*h*_] and *C *[ *I*_0,*n*_,*I*_*h*,*m*_] = *A* ⊗ *B *[ *I*_0,*k*_,*I*_*h*,*m*_].

2. For every 0 ≤ *h* < *n*, *C *[ *I*_0,*h*_,*I*_0,*m*_] = *A *[ *I*_0,*h*_,*I*_0,*k*_] ⊗ *B* and *C *[ *I*_*h*,*n*_,*I*_0,*m*_] = *A*[ *I*_*h*,*n*_,*I*_0,*k*_] ⊗ *B*.

3. For every 0 ≤ *h* < *k*, *C *= min {*A *[ *I*_0,*n*_,*I*_0,*h*_] ⊗ *B *[ *I*_0,*h*_,*I*_0,*m*_],*A *[ *I*_0,*n*_,*I*_*h*,*k*_] ⊗ *B *[ *I*_*h*,*k*_,*I*_0,*m*_]}.

### EDDC expressed via min-plus vector multiplications

The key observation that enables a further improvement of the worst-case bounds of EDDC is that Equations 3, 6, 8, and 9 can be expressed in terms of *min-plus vector multiplications*. Under the assumption that all solutions to sub-instances appearing on the right-hand side of the equations are computed and stored in the corresponding entries, these equations can be written as follows:

(10) $$\;\begin{array}{l} \begin{array}{ll} ed^{\prime }\left(\alpha ,t_{l,j}\right)\; & \overset{\text{Eq.3}}{=}\;\text{min}\;\left\{\left(\begin{array}{l} T^{\alpha }[\;l,h]+T^{\varepsilon }[\;h,j],\\ T^{\varepsilon }[\;l,h]+T^{\alpha }[\;h,j],\\ \text{dup}(\alpha )\;+\;T^{\alpha }[\;l,h]+T^{\alpha }[\;h,j] \end{array},\;\right|\begin{array}{l} \;l\;<\;h\;<\;j, \end{array},\;\right\}\\ & \;=\;\text{min}\;\left\{\begin{array}{l} T^{\alpha }[\;l,I_{l+1,j}]⊗T^{\varepsilon }[\;I_{l+1,j},j],\\ T^{\varepsilon }\left[\;l,I_{l+1,j}\right]⊗T^{\alpha }[\;I_{l+1,j},j],\\ \text{dup}(\alpha )+T^{\alpha }[\;l,I_{l+1,j}]⊗T^{\alpha }[\;I_{l+1,j},j] \end{array}\;\right\}\;, \end{array} \end{array}$$

(11) $$\;\begin{array}{l} \begin{array}{ll} ed^{\prime }\left(s_{k,i},\alpha \right)\; & \overset{\text{Eq.6}}{=}\;\text{min}\;\left\{\left(\begin{array}{l} S^{\alpha }[\;k,h]\;+S^{\varepsilon }[\;h,i],\\ S^{\varepsilon }[\;k,h]\;+S^{\alpha }[\;h,i],\\ S^{\alpha }[\;k,h]\;+S^{\alpha }[\;h,i]\;+\;\text{cont}(\alpha ) \end{array},\;\right|\begin{array}{l} \;k\;<\;h\;<\;i \end{array},\;\right\}\\ & \;=\;\text{min}\;\left\{\begin{array}{l} S^{\alpha }[\;k,I_{k+1,i}]⊗S^{\varepsilon }[\;I_{k+1,i},i],\\ S^{\varepsilon }[\;k,I_{k+1,i}]⊗S^{\alpha }[\;I_{k+1,i},i],\\ S^{\alpha }[\;k,I_{k+1,i}]⊗S^{\alpha }[\;I_{k+1,i},i]+\;\text{cont}(\alpha ) \end{array},\;\right\}, \end{array} \end{array}$$

(12) $$\;\begin{array}{l} \begin{array}{ll} \text{ed}\left(s_{0,i},t_{0,j}\right) & \overset{\text{Eq.8}}{=}\;\text{min}\;\left\{\begin{array}{l} S^{\alpha }[\;0,i]+T^{\alpha }[\;0,j],\\ \text{ED}T^{\;\alpha }[\;k,j]+S^{\alpha }[\;k,i]\; \end{array}\;\left|\begin{array}{l} \;0<k<i\;\\ \;\alpha \in \Sigma \end{array},\;\right)\right\}\\ & \;=\;\text{min}\;\left\{\left(\begin{array}{l} S^{\alpha }[\;0,i]+T^{\alpha }[\;0,j],\\ \text{tr}(S^{\alpha })[\;i,I_{1,i}]⊗\text{ED}T^{\;\alpha }[\;I_{1,i},j] \end{array},\;\right|\alpha \in \Sigma \;\right\}, \end{array} \end{array}$$

(13) $$\;\begin{array}{ll} \text{ed}t^{\alpha }\left(s_{0,i},t_{0,j}\right) & \overset{\text{Eq.9}}{=}\;\text{min}\;\left\{\text{ED}[\;i,l]+T^{\alpha }[\;l,j]\;|\;0<l<j\right\}\\ & \;=\;\text{ED}\;[\;i,I_{1,j}]⊗T^{\alpha }[\;I_{1,j},j]. \end{array}$$

### The algorithm

The new algorithm has the same two stages as the baseline algorithm. It can be observed that the computation of all matrices of the forms *S*^′*α*^, *S*^*α*^, *S*^*ε*^, *T*^′*α*^, *T*^*α*^, and *T*^*ε*^ performed in Stage 1 of the baseline algorithm adhere to the *Inside-VMT requirements* as given in Definition 1 in [11]. The application of the generic *Inside-VMT algorithm *[11] to this computation is immediate, and therefore we focus only on adapting the method to the computation of matrices of the form *EDT*^*α*^ and *ED* conducted in Stage 2 of the baseline algorithm.

**Figure 4:**
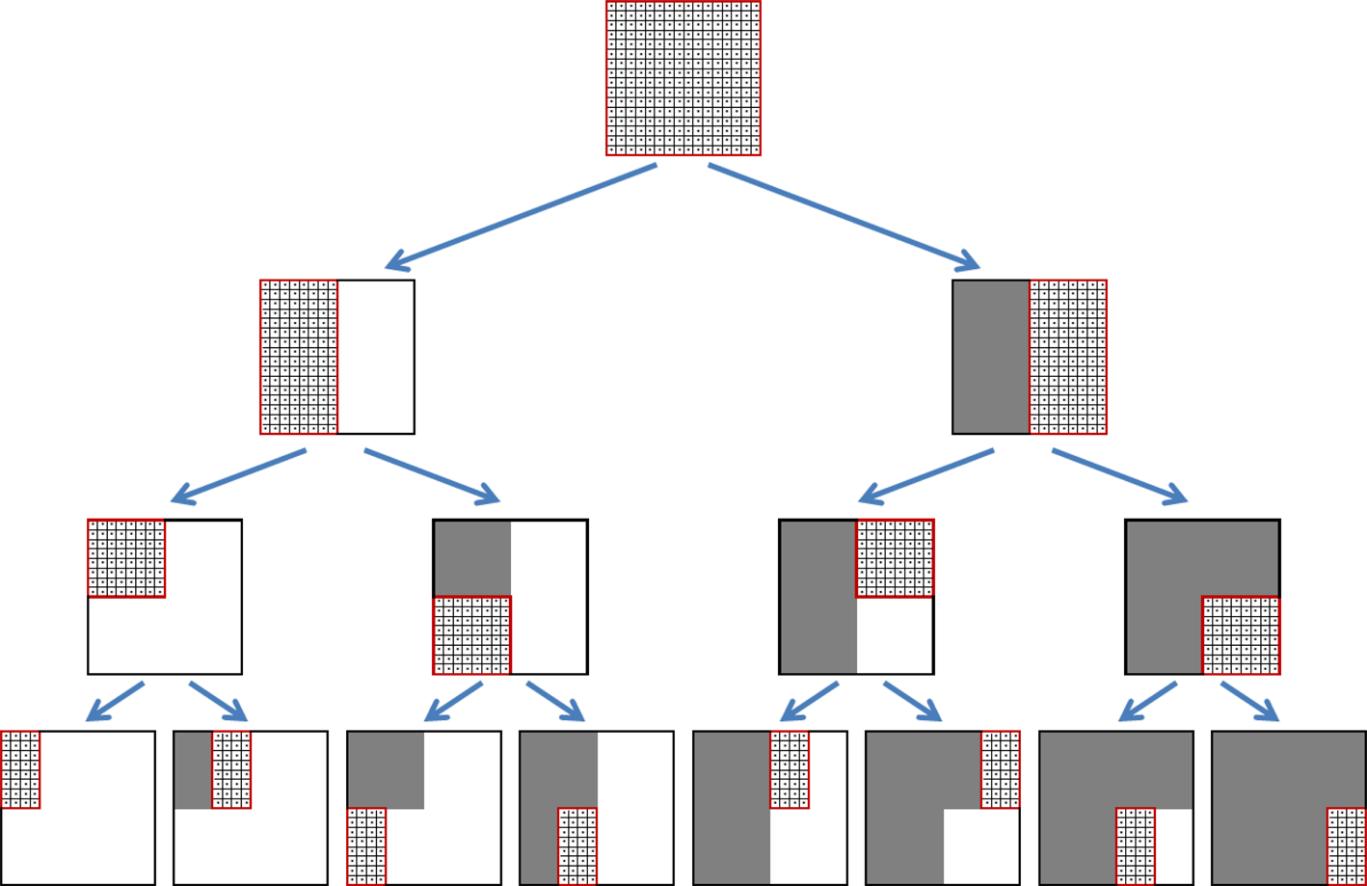
**The tree of recursive calls to COMPUTE-MATRIX.** Each call over a region containing more than one entry partitions the region either vertically or horizontally, and performs two recursive calls over the two sub-regions. After the first call was performed (the left or top sub-region for vertical or horizontal partition, respectively), a matrix multiplication involving the computed region is computed in order to meet the precondition required for the computation of the sibling region.

After allocating all dynamic programming matrices and performing Stage 1 of the algorithm, the COMPUTEMATRIX procedure is used for implementing Stage 2 (see Algorithm 2 and Figure 4). This is a divide-and-conquer recursive procedure that accepts a region *I* × *J* and computes the values in all entries of *ED* and *EDT*^*α*^ within the region. The procedure partitions the given region into two parts and performs recursive calls on each part. In order to maintain a required precondition, the procedure applies min-plus matrix multiplication subroutines between recursive calls. The correctness proof of Algorithm 2 appears in Appendix “Correctness of Algorithm 2”.

#### **Algorithm 2 MATRIX-EDDC(*s*,** ***t*)**

### Time complexity analysis

The time complexity of Algorithm 2 can be established by an identical analysis to that of the Inside-VMT algorithm of [11] (see Section 3.3.1 of [11]). For completeness, we repeat this analysis here for Stage 2 of the computation, where the time complexity of Stage 1 can be inferred similarly. The complexity is expressed as a function of the bound *MP *(*n*) over the running time of a min-plus multiplication of two *n* × *n* matrices. Note that *MP *(*n*) = *Ω *(*n*^2^), as the input and output matrices of the computation contain *O *(*n*^2^) entries. We assume here that *MP *(*n*) = *Ω *(*n*^2+*δ*^) for some constant 0 < *δ* ≤ 1, which is true for the current best bound over *MP *(*n*) [12]. In some of the expressions developed below, we avoid the “big *O*” notation and give explicit bounds over the number of operations, as constant factors that may be hidden due to this notation cannot be ignored in the analysis.

The initialization time of Stage 2 is dominated by the matrix multiplications and entry-wise min operations performed in line 4 of Algorithm 2. This initialization performs 2|*Σ*| multiplications of matrices of dimensions (*n*-1)×1 with matrices of dimensions 1×(*n* - 1), which can naively be implemented in *O *(|*Σ*|*n*^2^) time, and an entry-wise min operation over a set containing |*Σ*| matrices of dimensions (*n* - 1) × (*n* - 1), which is also implemented in *O *(|*Σ*|*n*^2^) time.

The computation of the remaining entries in *ED* and *EDT*^*α*^ matrices is done within recursive calls to the COMPUTE-MATRIX procedure. Observe that when COMPUTE-MATRIX is called over a region of dimensions *r* × *r* for some even integer *r* ≥ 2, the procedure applies a vertical partitioning and performs two recursive calls over regions of dimensions $r\times \frac{r}{2}$ (lines 6 and 8). For a call over a region of dimensions $r\times \frac{r}{2}$, the procedure applies a horizontal partitioning and performs two recursive calls over regions of dimensions $\frac{r}{2}\times \frac{r}{2}$ (lines 11 and 13). For simplicity, assume that *n* - 1 = 2^*p*^ for some integer *p* ≥ 0, and thus it follows that the dimensions of all regions occurring as inputs in recursive calls are either 1×1, or of the form *r* × *r* or $r\times \frac{r}{2}$ for some even integer *r*. Denote by *T *(*x* × *y*) an upper bound over the number of operations conducted when applying COMPUTE-MATRIX over a region of dimensions *x* × *y*.

From line 2 of the procedure, *T *(1 × 1) = *O *(|*Σ*|). Consider a region of dimensions *r* × *r* for an even integer *r* ≥ 2. For such a region, the code in lines 5-8 of COMPUTE-MATRIX is executed. In order to implement line 7 for some *α* ∈ *Σ*, it is necessary to compute first a min-plus matrix multiplication *C* = *A* ⊗ *B*, where the matrix *A* = *ED *[ *I*_*i*,*k*_,*I*_*j*,*h*_] is of dimensions $r\times \frac{r}{2}$, the matrix *B* = *T*^*α*^[ *I*_*j*,*h*_,*I*_*h*,*l*_] is of dimensions $\frac{r}{2}\times \frac{r}{2}$, and the resulting matrix *C* is of dimensions $r\times \frac{r}{2}$. Due to Observation 1, it is possible to compute independently the upper and lower halves of *C*, where $C[\;I_{0,\frac{r}{2}},I_{0,\frac{r}{2}}]=A[\;I_{0,\frac{r}{2}},I_{0,\frac{r}{2}}]⊗B$ and $C[\;I_{\frac{r}{2},r},I_{0,\frac{r}{2}}]=A[\;I_{\frac{r}{2},r},I_{0,\frac{r}{2}}]⊗B$. The time required to conduct this computation is $2\text{MP}(\frac{r}{2})$. Then, it is required to compute *min *{*EDT *^*α *^[ *I*_*i*,*k*_, *I*_*h*,*l*_], *C*} and to update *EDT *^*α *^[ *I*_*i*,*k*_,*I*_*h*,*l*_] to be the result of this operation, a computation which requires at most *cr *^2^ operations for some constant *c*. Since line 7 is computed for every *α* ∈ *Σ*, the total number of applied operations due to this line is at most $|\Sigma |\left(2\text{MP}(\frac{r}{2})+cr^{2}\right)$. Besides line 7, two recursive calls are made in lines 6 and 8 over regions of dimensions $r\times \frac{r}{2}$, and therefore we get

$$T(r\times r)\leq 2T\left(r\times \frac{r}{2}\right)+|\Sigma |\left(2\text{MP}\left(\frac{r}{2}\right)+cr^{2}\right).$$

When the procedure is called over a region of dimensions $r\times \frac{r}{2}$, the code in lines 10-13 is executed. Similarly as above, it can be shown that the computation in line 12 requires at most $|\Sigma |\left(\text{MP}(\frac{r}{2})+\frac{cr^{2}}{4}\right)$ operations, and due to the two recursive calls in lines 11 and 13 over regions of dimensions $\frac{r}{2}\times \frac{r}{2}$, we get

$$T\left(r\times \frac{r}{2}\right)\leq 2T\left(\frac{r}{2}\times \frac{r}{2}\right)+|\Sigma |\left(\text{MP}\left(\frac{r}{2}\right)+\frac{cr^{2}}{4}\right).$$

Therefore,

$$T(r\times r)\leq 4T\left(\frac{r}{2}\times \frac{r}{2}\right)+|\Sigma |\left(4\text{MP}\left(\frac{r}{2}\right)+\frac{3cr^{2}}{2}\right).$$

The explicit form of the above recursive equation can be established by the Master Theorem (under the assumption that *MP *(*n*) = *Ω *(*n*^2+*δ*^), see Chapter 4 in [16]), yielding the expression *T *(*r* × *r*) = *O *(|*Σ*|*MP *(*r*)). Thus, the time complexity of Stage 2 of the algorithm is *O *(|*Σ*|*MP *(*n*)). The time analysis of the Inside-VMT algorithm of [11], applied to implement Stage 1 of the algorithm yields the same bound of *O *(|*Σ*|*MP *(*n*)), and thus *O *(|*Σ*|*MP *(*n*)) is the time complexity of the entire algorithm. Using the currently asymptotically fastest algorithm for min-plus matrix multiplication [12]$\text{MP}(n)=\Theta \left(\frac{n^{3}\overset{3}{\text{log}}\text{log}n}{\overset{2}{\text{log}}n}\right)$, we get the currently best explicit time bound for EDDC of $O\left(\frac{|\Sigma |n^{3}\overset{3}{\text{log}}\text{log}n}{\overset{2}{\text{log}}n}\right)$.

## An online algorithm for EDDC using min-plus matrix-vector multiplication for discrete cost functions

In this section, we present an EDDC algorithm which is based on the general algorithm (given in Section “A matrix multiplication based algorithm for EDDC”) and improves its time complexity by a factor of *O *(log3 log*n*). This EDDC algorithm is intended for integer cost functions, but can also be applied to rational cost functions after they are scaled. It is an online algorithm; it can process the input strings letter by letter with a guaranteed low time bound for any prefix of the input. The EDDC algorithm presented in this section is based on a *D*-discrete matrix-vector min-plus multiplication algorithm we developed, which is generic and may be applied to other problems as well.

### *D*-discrete matrices and the EDDC problem with integer costs

Given a matrix of integers *A*_*n* × *m*_ and indices 1 ≤ *i* < *n* and 0 ≤ *j* < *n*, call the pair of entries *A *[ *i* - 1,*j*] and *A *[ *i*,*j*]* adjacent*. Let *D* = *I*_*a*,*b *_= [ *a*,*a* + 1,…,*b* - 1] be an integer interval for some integers *a* < *b*. Say that matrix *A* is *D*-discrete if for every pair of adjacent entries *A *[ *i* - 1,*j*] and *A *[ *i*,*j*], their difference *A *[ *i* - 1,*j*] - *A *[ *i*,*j*] is in *D*.

Consider the EDDC problem in the case of integer costs for all edit operations. In Lemma 1, we show that in this case, all matrix multiplications applied by Algorithm 2 are between *D*-discrete metrices, with respect to a certain integer interval *D*. This proof is similar to that of Masek and Paterson for simple edit distance [17]. This would allow conducting such matrix multiplications using a faster algorithm, described in Section “An efficient *D*-discrete min-plus matrix-vector multiplication algorithm”.

#### 

**Lemma 1.** *Given strings s and t and an integer cost function for EDDC, all matrix multiplications applied by Algorithm 2 are over D-discrete matrices, where D = I*_*a*,*b*_ *is determined according to the cost function by a=- max{del*(*α*) | *α* ∈ *Σ*} *and b = max*{*ins *(*α*) | *α* ∈ *Σ*} + 1.

The proof of Lemma 1 appears in Appendix “Proofs to lemmas corresponding to the EDDC algorithm for discrete cost functions”.

### *D*-discrete matrices and vectors

Here, we present some properties of *D*-discrete matrices and vectors that are similar to those previously observed in [14,17]. The following lemmas show that the set of *D*-discrete matrices is closed under the min-plus multiplication and entry-wise min operations. In what follows, let *D*=*I*_*a*,*b*_ be some integer interval. The proofs of the following lemmas appear in Appendix “Proofs to lemmas corresponding to the EDDC algorithm for discrete cost functions”.

#### 

**Lemma 2.** *Let X*,*Y and Z be matrices, such that X and Y contain only integer elements and Z *= *X* ⊗ *Y*. *If X is D-discrete, then is D-discrete*.

#### 

**Lemma 3.** *Let X*,*Y and Z be matrices, such that X and Y contain only integer elements and Z = min*{*X*,*Y*}. *If X and Y are D -discrete, then Z is D-discrete*.

The following lemma implies that when the absolute difference between the first elements of two *q*-length *D*-discrete vectors *x* and *y* is sufficiently large, one of the vectors can be immediately taken as the result of the min(*x*,*y*) operation.

#### 

**Lemma 4.** *Let x =*(*x*_0_,…,*x*_*q *- 1_)* and y = *(*y*_0_,…,*y*_*q *- 1_)* be two q-length D-discrete vectors for some q *> 0. *If y*_0 _- *x*_0_ ≥ *q *|*D*|*, then min*(*x*,*y*) =  *x*.

In what follows, fix an integer *q* > 1. Let *x* = (*x*_0_,*x*_1_,…,*x*_*q*-1_) be a *q*-length *D*-discrete vector. By definition, for every 0<*i* < *q*, *x*_*i*-1_-*x*_*i*_ is an integer within *D*, and so *x*_*i*-1_-*x*_*i*_ - *a* is an integer within the interval *I*_0,*b*-*a*_ = *I*_0,|*D*|_. Therefore, the series *x*_0_ - *x*_1_ - *a*,*x*_1_ - *x*_2_ - *a*,…,*x*_*q*-2_-*x*_*q*-1_-*a* can be thought of as a series of *q*-1 digits in a |*D*|-base representation of an integer $\Delta x=\underset{0\leq i<q-1}{\sum }|D{|}^{i}(x_{i}-x_{i+1}-a)$, where 0 ≤ *Δ* *x* < |*D*|^*q*-1^. The *Δ-encoding* of *x* is defined to be the pair of integers (*x*_0_,*Δ* *x*). We write *x* = (*x*_0_,*Δ* *x*) to indicate that (*x*_0_,*Δ* *x*) is the *Δ*-encoding of *x*, where *x*_0_ is called the *offset* of *x* and *Δ x* is called the *canonical index* of *x*. Note that for two *q*-length *D*-discrete vectors *x* = (*x*_0_,*Δ* *x*) and *y* = (*y*_0_,*Δ* *y*), *Δ* *x* = *Δy* if and only if for every 0 ≤ *i* < *q*, *x*_*i*_ - *y*_*i*_ = *c* for some constant *c*. In particular, *x* and *y* share the same *Δ*-encoding if and only if they are identical. Call a *D*-discrete vector of the form *x* = (0,*Δ* *x*) (with an offset *x*_0_ = 0) a *canonical*vvector.

The next observations show that both operations of entry-wise min and min-plus multiplication, with respect to *D*-discrete matrices and vectors, can be expressed via canonical vectors.

#### 

**Observation 2.** *Let x *= (*x*_0_,*Δ x*), *y* = (*y*_0_,*Δ y*), *and z *= (*z*_0_,*Δ z*)* be q-length D-discrete vectors such that z = min *(*x*,*y*). *Then, for every number c it holds that min((x*_0_ - *c*,*Δ x*),(*y*_0_ - *c*,*Δ y*)) = (*z*_0_ - *c*,*Δ z*). *In particular, min((0,Δ x*),(*y*_0_ - *x*_0_,*Δy*)) = (*z*_0_ - *x*_0_,*Δ z*).

#### 

**Observation 3.** *LetB*_*q* × *q*_ *be a D-discrete matrix, x = (0,Δ  x*)* a q-length canonical D-discrete vector, and y =(y*_0_,*Δ y*)* a q-length D-discrete vector, such that B* ⊗ *x *= *y*. *Then, for any number c it holds that B* ⊗ (*c*,*Δ x*) = (*y*_0_ + *c*,*Δ y*).

### An efficient *D*-discrete min-plus matrix-vector multiplication algorithm

Let *A*_*n*×*m*_ be a *D*-discrete matrix, and fix a constant $\frac{1}{\underset{\left|D\right|}{\text{log}}(n+m)}<\lambda <1$. We give an algorithm for min-plus *D*-discrete matrix-vector multiplication that, after preprocessing *A* in $O\left(\frac{\text{nm}{\left(n+m\right)}^{\lambda }}{\left|D\right|}\right)$ time and $O\left(\frac{\text{nm}{\left(n+m\right)}^{\lambda }}{\left|D|\lambda^{2}\overset{2}{\underset{\left|D\right|}{\text{log}}}(n+m\right)}\right)$ space, computes *A* ⊗ *x* for any *m*-length *D*-discrete vector *x* in $O\left(\frac{\text{nm}}{\lambda^{2}\overset{2}{\underset{\left|D\right|}{\text{log}}}(n+m)}\right)$ time under the RAM computational model. Our algorithm is an adaptation of Williams’ algorithm [13] for finite semiring matrix-vector multiplications, with some notions similar to Frid and Gusfield’s acceleration technique for RNA folding [14]. It follows the concept of the *Four-Russians Algorithm *[18] (see also [14,17,19]), i.e. preprocessing reoccurring computations, tabulating their results in lookup tables, and retrieving such results in order to accelerate the general computation.

Specifically, the algorithm stores preprocessed computations of two kinds: matrix-vector min-plus multiplications, and vector entry-wise minima, where vectors and matrices are of *q*-length and of *q* × *q* dimensions, respectively, for *q* = ⌊ *λ* log|*D*|(*n* + *m*) ⌋. For conducting this preprocessing, we will assume that |*D*| ≤ *n* + *m*, otherwise *q *= 0 and the multiplication cannot be accelerated using the suggested method. In addition, for simplicity of the analysis we assume that *q*^3^ ≤ min(*n*,*m*). If this does not hold, a multiplication of the form *A* ⊗ *x* can be naively computed in the relatively efficient time complexity of $O\left(\text{max}(n,m)\overset{3}{\underset{\left|D\right|}{\text{log}}}\left(n+m\right)\right)$. The space complexity of the preprocessing phase is higher than the *O *(*nm*) space complexity of the standard multiplication algorithm and depends on the constant *λ*, ranging between *O*(*nm*|*D*|) and $O\left(\frac{\text{nm}(n+m)}{\overset{2}{\underset{\left|D\right|}{\text{log}}}(n+m)}\right)$ for *λ* values between $\frac{1}{\underset{\left|D\right|}{\text{log}}(n+m)}$ and 1, correspondingly. The lower bound $\frac{1}{\underset{\left|D\right|}{\text{log}}(n+m)}$ for *λ* is chosen so that the time complexity $O\left(\frac{\text{nm}}{\lambda^{2}\overset{2}{\underset{\left|D\right|}{\text{log}}}(n+m)}\right)$ of matrix-vector multiplications involving the preprocessed matrix would be better than the naive time complexity *O *(*nm*).

#### ***Preprocessing of matrix-vector ⊗ computations***

Let $n^{\prime }=q\left\lfloor \frac{n}{q}\right\rfloor$ and $m^{\prime }=q\left\lfloor \frac{m}{q}\right\rfloor$, and note that 0 ≤ *n* - *n*^′^ < *q* and 0 ≤ *m* - *m*^′^ < *q*. Let *Q*_*k*_ denote the *q*-length integer interval *Q*_*k*_ = [ *kq*,*kq* + 1,…,(*k* + 1) *q* - 1]. The sub-matrix $A^{\prime }=A\left[I_{0,n^{\prime }},I_{0,m^{\prime }}\right]$ is decomposed into $\frac{n^{\prime }m^{\prime }}{q^{2}}$ blocks *B*_*i*,*j*_ = *A*[ *Q*_*i*_,*Q*_*j*_] where $i=0,1,\ldots ,\frac{n^{\prime }}{q}-1$ and $j=0,1,\ldots ,\frac{m^{\prime }}{q}-1$. For each block *B*, a corresponding lookup table *MUL*_*B*_ is created, which tabulates min-plus multiplications between *B* and all canonical *q*-length *D*-discrete vectors. For the canonical vector *x* = (0,*Δx*), the result *y* = *B *⊗ *x* is stored in the entry *MUL*_*B *_[ *Δ x*] by its encoding (*y*_0_,*Δ y*) (by Lemma 2, *y* is also *D*-discrete and thus can be encoded accordingly).

The multiplication of a *q* × *q* block with a *q*-length vector can be done in *O *(*q*^2^) time in a straightforward manner and the encoding of the resulting *q*-length vector requires additional *O *(*q*) time. There are $\frac{n^{\prime }m^{\prime }}{q^{2}}$ blocks in the decomposition of *A*^′^, each is multiplied by |*D*|^*q*-1^ canonical vectors, and so the total time required for computing these multiplications is $O\left(q^{2}|D{|}^{q-1}\frac{\text{nm}}{q^{2}}\right)=O\left(|D{|}^{q-1}\text{nm}\right)=O\left(\frac{\text{nm}{\left(n+m\right)}^{\lambda }}{\left|D\right|}\right)$.

Let (*y*_0_,*Δ y*) be the *Δ *- encoding of some result *y* = *B *⊗ *x* computed in the preprocessing of *A*^′^ as described above. Note that *y*_0_ = min 0 ≤ *i* < *q *{*B *[ 0,*i*] + *x *[ *i*]} ≤ min 0 ≤ *i* < *q *{2 max(*B *[ 0,*i*],*x*[ *i*])}. Therefore, the number of bits in the binary representation of *y*_0_ is at most one plus the maximum number of bits required for the representations of *B *[ 0,*i*] and *x *[ *i*] for some 0 ≤ *i *< *q*. Also, note that $0\leq \Delta y<|D{|}^{q-1}=\frac{{\left(n+m\right)}^{\lambda }}{\left|D\right|}$, and *Δ y* can be represented in *O *(log(*n* + *m*)) bits. Thus, under the RAM computational model assumptions, each such encoding (*y*_0_,*Δ y*) requires *O *(1) space units and can be written and read in a constant time, and therefore the overall space complexity for maintaining all *MUL*_*B*_ tables is $O\left(\frac{|D{|}^{q-1}\text{nm}}{q^{2}}\right)=O\left(\frac{\text{nm}{\left(n+m\right)}^{\lambda }}{\left|D|\lambda^{2}\overset{2}{\underset{\left|D\right|}{\text{log}}}(n+m\right)}\right)$. In addition, given a canonical index *Δ x*, it is possible to retrieve the encoding (*y*_0_,*Δ y*) = *B *⊗ (0,*Δ x*) stored in the entry *MUL*_*B *_[ *Δ x*] in a constant time.

Let *x* = (*x*_0_,*Δ x*) be some (not necessarily canonical) *q*-length *D*-discrete vector, for which we wish to compute *B* ⊗ *x*. Due to Observation 3, the multiplication result can be obtained in constant time by retrieving (*y*_0_,*Δ y*) = *MUL*_*B *_[ *Δ x*], and returning the encoding (*y *_0_ + *x*_0_,*Δ y*).

#### ***Preprocessing of vector entry-wise min computations***

The algorithm constructs a lookup table *MIN*, storing entry-wise min calculations between every canonical *q*-length *D*-discrete vector *x* = (0,*Δ x*) and every *q*-length *D*-discrete vector *y* = (*y*_0_,*Δ y*) such that *abs *(*y *_0_) < *q*|*D*| (here *abs *(*y*_0_) denotes the absolute value of *y*_0_). For every such *x* and *y*, the table entry *MIN *[ *Δx*,*y*_0_,*Δ y*] stores the *Δ *- encoding (*z*_0_,*Δ z*) of the vector *z* = min (*x*,*y*) (due to Lemma 3, *z* is *D*-discrete and can be encoded accordingly). There are $O\left(q|D||D{|}^{2(q-1)}\right)=O\left(\frac{{\left(n+m\right)}^{2\lambda }\lambda \underset{\left|D\right|}{\text{log}}(n+m)}{\left|D\right|}\right)$ entries in the table *MIN*, and each entry can be computed in *O*(*q*) = *O *(*λ* log|*D*|(*n* + *m*)) time. Thus, the computation of all entries in *MIN* requires $O\left(\frac{{\left(n+m\right)}^{2\lambda }\lambda^{2}\overset{2}{\underset{\left|D\right|}{\text{log}}}(n+m)}{\left|D\right|}\right)$ time, and the table occupies $O\left(\frac{{\left(n+m\right)}^{2\lambda }\lambda \underset{\left|D\right|}{\text{log}}(n+m)}{\left|D\right|}\right)$ space.

Given two encoded *q*-length *D*-discrete vectors *x* = (*x*_0_,*Δ x*) and *y* = (*y*_0_,*Δ y*), the encoding (*z*_0_,*Δ z*) of the vector *z* = min(*x*,*y*) can now be obtained in a constant time as follows: (*z*_0_,*Δ z*) = (*x*_0_,*Δx*) if *y*_0_ - *x*_0_ ≥ *q *|*D*| or (*z*_0_,*Δ z*) = (*y*_0_,*Δ y*) if *x*_0_ - *y*_0_ ≥ *q *|*D*|, due to Lemma 4. Otherwise, |*y*_0_ - *x*_0_| < *q *|*D*|, and for the vectors *x*^′^ = (0,*Δx*), *y *^′^ = (*y*_0_ - *x*_0_,*Δ y*), and $z^{\prime }=\left(z_{0}^{\prime },\Delta z^{\prime }\right)=\text{min}(x^{\prime },y^{\prime })$, we have that (*z*^′^,*Δ z*^′^) = *MIN *[ *Δx*,*y*_0_ - *x*_0_,*Δ y*]. From Observation 2, $\left(z_{0},\Delta z\right)=\left(z_{0}^{\prime }+x_{0},\Delta z^{\prime }\right)$.

#### ***Computing matrix-vector multiplications***

Given an *m*-length *D*-discrete vector *x* and assuming the preprocessing of matrix *A*_*n* × *m*_ was preformed as described above, we next explain how to efficiently compute the vector *y* = *A* ⊗ *x*. Note that *y* is an *n*-length *D*-discrete vector, due to Lemma 2.

**Figure 5:**
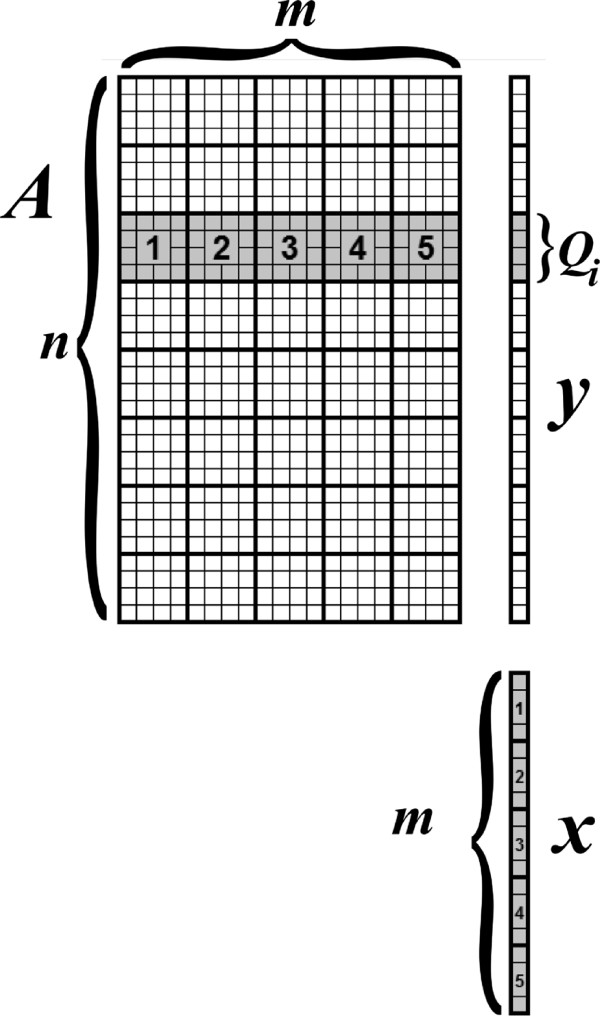
The computation of a q-length segment in a multiplication of a D-discrete matrix with a D-discrete vector.

Our algorithm computes first the multiplication $y^{\prime }=A^{\prime }⊗x[I_{0,m^{\prime }}]$ in parts of length *q*. First, for every $0\leq j<\frac{m^{\prime }}{q}$, the algorithm computes the encoding $\left(x_{\;0}^{\;j},\Delta x^{\;j}\right)$ of the sub-vector *x*^*j*^ = *x *[ *Q*_*j*_] of *x*. These encodings can be obtained in a total time of *O *(*m*). Then, for every $0\leq i<\frac{n^{\prime }}{q}$, the encoding $\left(y_{\;0}^{i},\Delta y^{i}\right)$ of the sub-vector $y^{i}=A^{\prime }\left[\;Q_{i},I_{0,m^{\prime }}\right]⊗x\left[\;I_{0,m^{\prime }}\right]$ of *y*^′^ is computed independently of the other sub-vectors of *y*^′^. By definition (see Figure 5),

$$\begin{array}{ll} y^{i} & =\text{min}\left\{A^{\prime }\left[\;Q_{i},Q_{j}\right]⊗x\left[\;Q_{j}\right]\;|\;0\leq j<\frac{m^{\prime }}{q}\right\}\\ & =\text{min}\left\{B_{i,j}⊗x^{\;j}\;|\;0\leq j<\frac{m^{\prime }}{q}\right\}. \end{array}$$

The encoded result $\left(z_{\;\;0}^{i,j},\Delta z^{i,j}\right)$ of each multiplication *z*^*i*,*j*^ = *B*_*i*,*j*_ ⊗ *x*^*j*^ can be obtained in a constant time as explained in Section “Preprocessing of matrix-vector ⊗ computations”. As there are $\frac{m^{\prime }}{q}$ such terms to compute with respect to *y*^*i*^, their total computation time is $O\left(\frac{m}{q}\right)$. In addition, the entry-wise min over all these terms can be computed by initializing $\left(y_{\;0}^{i},\Delta y^{i}\right)\leftarrow \left(z_{\;\;\;0}^{i,0},\Delta z^{i,0}\right)$, and iteratively updating $\left(y_{\;0}^{i},\Delta y^{i}\right)\leftarrow \text{min}\left(\left(\overset{i}{\underset{\;0}{y}},\Delta y^{i}\right),\left(\overset{i,j}{\underset{\;\;0}{z}},\Delta z^{i,j}\right)\right)$ for all $0<j<\frac{m^{\prime }}{q}$. Each such update is computed in a constant time as described in Section “Preprocessing of vector entry-wise min computations”, and so the encoding of a single segment *y*^*i*^ in *y*^′^ is computed in a total time of $O\left(\frac{m}{q}\right)$, and the encodings of all $O\left(\frac{n}{q}\right)$ such segments are computed in $O\left(\frac{\text{nm}}{q^{2}}\right)$ time. Decoding all encoded vectors *y*^*i*^ can be done in additional *O *(*n*) operations, obtaining an explicit form of *y*^′^ in a total time of $O\left(\frac{\text{nm}}{q^{2}}\right)$.

Let $y^{\prime \prime }=A\left[I_{0,n^{\prime }},I_{m^{\prime },m}\right]⊗x\left[I_{m^{\prime },m}\right]$, where from Observation 1, $y\left[I_{0,n^{\prime }}\right]=\text{min}\left(y^{\prime },y^{\prime \prime }\right)$. The computation of *y*^′′^ can be conducted in *O *(*nq*) time in a straightforward manner, and the computation of min(*y*^′^,*y*^′′^) requires additional *O *(*n*) time. In addition, $y\left[I_{n^{\prime },n}\right]=A\left[I_{n^{\prime },n},I_{0,m}\right]⊗x$, where this computation can be done naively in *O *(*mq*) time, and so the overall running time for computing *y * is $O\left(\frac{\text{nm}}{q^{2}}+\text{nq}+\text{mq}\right)=O\left(\frac{\text{nm}}{q^{2}}\right)=O\left(\frac{\text{nm}}{\lambda^{2}\overset{2}{\underset{\left|D\right|}{\text{log}}}(n+m)}\right)$.

The above matrix-vector min-plus multiplication algorithm can be used as a fast square matrix-matrix multiplication algorithm in a straightforward manner. For two *D*-discrete matrices *A*_*n* × *n*_ and *B*_*n* × *n*_, the computation of *C* = *A* ⊗ *B* can be conducted by first preprocessing *A* as described in Sections “Preprocessing of matrix-vector ⊗ computations” in $O\left(\frac{n^{2+\lambda }}{\left|D\right|}\right)$ time and $O\left(\frac{n^{2+\lambda }}{|D|\lambda^{2}\overset{2}{\underset{\left|D\right|}{\text{log}}}n}\right)$ space, and then computing each column *j* of *C* independently by multiplying *A* with the *j*-th column of *B*, in $O\left(\frac{n^{2}}{\lambda^{2}\overset{2}{\underset{\left|D\right|}{\text{log}}}n}\right)$ time as explained above. The total computation time of all *n* columns of *C* is therefore $O\left(\frac{n^{3}}{\lambda^{2}\overset{2}{\underset{\left|D\right|}{\text{log}}}n}\right)$.

### Online preprocessing of *D*-discrete matrices

In the previous section, we assumed the settings in which a *D*-discrete matrix is given, and that it is preprocessed once prior to any multiplication operation. Next, we describe how to maintain the required lookup tables for the case the input matrix is dynamic, acquiring additional rows and columns. Consider a streaming computational model, which begins with an initial empty matrix $A_{0\times 0}^{0}$. In each step *r*, the current matrix $A_{n_{r}\times m_{r}}^{r}$ is obtained from the previous matrix $A_{n_{r-1}\times m_{r-1}}^{r-1}$ by either adding an *m*_*r*-1_-length vector as the last row or adding an *n*_*r*-1_-length vector as the last column in the matrix. Note that *n*_*r*_ + *m*_*r*_ = *r*, and therefore the preprocessing block length corresponding to *A*^*r*^ is *q* = ⌊ *λ* log|*D*|(*n*_*r*_ + *m*_*r*_) ⌋ = ⌊ *λ* log|*D*|(*r*)⌋. For the purpose of this analysis, we assume that *λ* ≤ 0.5 (note that this does not limit the asymptotic upper bounds of the running time). This assumption implies the following inequality

(14) $$|D{|}^{\frac{1}{\lambda }}\geq 2^{2}=4.$$

Lookup tables corresponding to intermediate matrices along the series can be maintained as follows. Let *r*_0_ and *r*_1_ be the smallest integers such that the block sizes corresponding to $A^{r_{0}}$ and $A^{r_{1}}$ are *q* and *q* + 1, respectively. Assume that upon reaching $A^{r_{0}}$ in the matrix sequence, all required lookup tables with respect to $A^{r_{0}}$ are already computed. Along the series of steps *r*_0_,*r*_0_ + 1,…,*r*_1_, we distribute two kinds of computations: (1) new *MUL*_*B*_ tables for accumulated *q* × *q* blocks in matrices *A*^*r*^ for *r*_0_ ≤ *r* < *r*_1_, and (2) a new *MIN* table, as well as new *MUL*_*B*_ tables, with respect to block length *q* + 1.

**(1) Computing *MUL***_***B ***_**tables for accumulated *q × q *** **blocks.** Assume that for some *r*_0_ ≤ *r* < *r*_1_, a column was added to the matrix at step *r* so that the number of columns *m*_*r*_ in the intermediate matrix *A*^*r*^ is divisible by *q*. Thus, at most $\frac{n_{r}}{q}\leq \frac{r}{q}$ new *q* × *q* complete blocks are now available for preprocessing. The computation of lookup tables of the form *MUL*_*B*_ corresponding to these new blocks will be equally distributed along the series of *q* consecutive steps *r*,*r* + 1,…,*r* + *q* - 1, during which it is guaranteed that no column addition would introduce new complete *q* × *q* blocks in the matrix. As shown in Section “Preprocessing of matrix-vector ⊗ computations”, the time required for processing a single *q* × *q* block is *O *(*q*^2^|*D*|^*q*-1^), and so the total time for processing all $O\left(\frac{r}{q}\right)$ blocks is *O *(*qr *|*D*|^*q*-1^). Thus, in each step among the *q* steps, there is a need to perform $O\left(r|D{|}^{q-1}\right)=O\left(\frac{r^{1+\lambda }}{\left|D\right|}\right)$ operations due to these computations. Symmetrically, computing lookup tables corresponding to new blocks added due to the accumulation of rows can be performed by conducting $O\left(\frac{r^{1+\lambda }}{\left|D\right|}\right)$ operations per step *r*.

**(2) Computing a new *MIN *** **lookup table and new *MUL***_***B ***_**tables with respect to block length *q + 1*.** By the selection of *r*_0_ and *r*_1_, *q* - 1 = ⌊ *λ *log|*D*|(*r*_0_ - 1) ⌋ > *λ* log|*D*|(*r*_0_ - 1) - 1, and *q* + 1= ⌊ *λ* log|*D*|(*r*_1_) ⌋ ≤ *λ* log|*D*|(*r*_1_). Therefore, $\underset{\left|D\right|}{\text{log}}(r_{1})>\underset{\left|D\right|}{\text{log}}(r_{0}-1)+\frac{1}{\lambda }$, and so $r_{1}>(r_{0}-1)|D{|}^{\frac{1}{\lambda }}\geq r_{0}\frac{|D{|}^{\frac{1}{\lambda }}}{2}\overset{\text{Eq.14}}{\geq }2r_{0}$. In particular, $r_{2}=\frac{r_{1}}{2}$ satisfies *r*_0_ < *r*_2_ < *r*_1_, and for every *r*_2_ ≤ *r* < *r*_1_ we have that *O *(*r*) = *O *(*r*_1_). The computation of the table *MIN* and tables of the form *MUL*_*B*_ with respect to block length *q* + 1 is distributed along the series of $\frac{r_{1}}{2}$ steps *r*_2_,*r*_2_ + 1,…,*r*_1_.

The new *MIN* table is computed independently from the specific input instance, and its overall computation time is $O\left(\frac{r_{1}^{2\lambda }\lambda^{2}\overset{2}{\underset{\left|D\right|}{\text{log}}}(r_{1})}{\left|D\right|}\right)$ (see Section “Preprocessing of vector entry-wise min computations”). By distributing this computation evenly along all *O *(*r*_1_) steps, the computation time required for each step *r*_2_ ≤ *r* < *r*_1_ is $O\left(\frac{r_{1}^{2\lambda -1}\lambda^{2}\overset{2}{\underset{\left|D\right|}{\text{log}}}(r_{1})}{\left|D\right|}\right)=O\left(\frac{r^{2\lambda -1}\lambda^{2}\overset{2}{\underset{\left|D\right|}{\text{log}}}(r)}{\left|D\right|}\right)$.

The *MUL*_*B*_ tables are computed similarly as done in (1), starting with (*q* + 1) × (*q* + 1) blocks already present in $A^{r_{2}}$, and continuing with blocks accumulated as the sequence progress. The overall preprocessing time of all these blocks is $O\left(\frac{r_{1}^{2+\lambda }}{\left|D\right|}\right)$ (see Section “Preprocessing of matrix-vector ⊗ computations”), and so the computation time required for each step *r*_2_ ≤ *r* < *r*_1_ is $O\left(\frac{r_{1}^{1+\lambda }}{\left|D\right|}\right)=O\left(\frac{r^{1+\lambda }}{\left|D\right|}\right)$.

All in all, the time complexity due to computations of (1) and (2) for each step *r*_0_ ≤ *r* < *r*_1_ is $O\left(\frac{r^{1+\lambda }}{\left|D\right|}\right)$. In particular, the overall time complexity of preprocessing the *n *- size prefix *A*^0^,*A*^1^,…,*A*^*n*^ of the streamed matrices is $O\left(\frac{n^{2+\lambda }}{\left|D\right|}\right)$.

### The EDDC algorithm based on efficient *D*-discrete min-plus matrix-vector multiplication

Consider the EDDC problem in cases where all edit operation costs are integers. As explained in Section “*D*-discrete matrices and the EDDC problem with integer costs*D*-discrete matrices and the EDDC problem with integercosts”, the EDDC DP tables can be considered *D*-discrete. This property allows for efficient min-plus square *D*-discrete matrix-vector multiplications, using the algorithm described in Section “An efficient *D*-discrete min-plus matrix-vector multiplication algorithm” to yield an $O\left(\frac{|\Sigma |n^{3}}{\overset{2}{\underset{\left|D\right|}{\text{log}}}n}\right)$ running time algorithm for EDDC. We next describe an online version of the algorithm, in which the letters of the input strings *s* and *t* are received in a streaming model.

Assume that some pair of prefixes *s*_0,*i*_ and *t*_0,*j*-1_ was already processed, and all entries in the DP matrices corresponding to these prefixes are computed. We explain how to update the tables in case where the next letter to arrive is the letter *t*_*j*-1_ in *t*, where the case in which the arriving letter is from *s* is symmetric. The DP matrices are *D*-discrete, and assume that lookup tables for efficient min-plus multiplications of these matrices are maintained as explained in the previous section. The addition of *t*_*j*-1_ requires updating all matrices of the forms *T*^*ε*^, *T*^*α*^, and *T*^′*α*^, for which the *j*-th row and column should be added. In addition, it is required to add the *j*-th column to matrices of the form *ED *and *EDT *^*α*^.

In the first stage, the algorithm computes rows and columns *j* in all matrices of the form *T*^′*α*^, *T*^*α*^, and *T*^*ε*^. The process is similar to the computation of these entries by the loop in lines 5 to 8 of Algorithm 1, with the following modification. Let *q*_*j*_ = ⌊ *λ* log|*D*|(2*j*) ⌋, and let $j^{\prime }=q_{j}\left\lfloor \frac{j}{q_{j}}\right\rfloor$. The algorithm first initializes the entries [ *j* - 1,*j*] in all these matrices with the corresponding base-case values. The column is partitioned to intervals of length *q*_*j*_, where as before *Q*_*k*_ denotes the interval $I_{kq_{j},(k+1)q_{j}}$. Once an interval *Q*_*k*_ is computed (i.e. the loop was executed with respect to index *l* = *kq*_*j*_), the *Δ* - encoding of the sub-vector *T*^*α*^[ *Q*_*k*_,*j*] is computed and kept for its later usage as lookup index. In addition, upon starting to compute the entries within an interval *Q*_*k*_ (i.e. when *l* = (*k* + 1)*q*_*j*_ - 1), the following multiplications are computed for every *α* ∈ *Σ*:

$$\;\begin{array}{ll} y^{\alpha ,k} & =T^{\alpha }\left[\;Q_{k},I_{q_{j}(k+1),j^{\prime }}\right]⊗T^{\alpha }\left[\;I_{q_{j}(k+1),j^{\prime }},j\right]\\ & \overset{\text{Obs.1}}{=}\text{min}\left\{T^{\alpha }\left[\;Q_{k},Q_{p}\right]⊗T^{\alpha }\left[\;Q_{p},j\right]\;|\;k<p<\frac{j^{\prime }}{q_{j}}\right\} \end{array}$$

Observe that all required entries for the computation of *y*^*α*,*k*^ are already computed and stored in *T*^*α*^, and that similarly as done in Section “Computing matrix-vector multiplications”, *y*^*α*,*k*^ can be computed by performing $O\left(\frac{j}{q_{j}}\right)$ constant time lookup table queries. After *y*^*α*,*k*^ is computed, *y*^*α*,*k *^[ *x*] contains the value min{*T*^*α *^[ *kq*_*j*_ + *x*,*h*] + *T*^*α *^[ *h*,*j*] | (*k* + 1)*q*_*j*_ ≤ *h* < *j*^′^}. Given *y*^*α*,*k *^[ *x*], the number of expressions that need to be examined in line 5 of the loop with respect to *l* = *kq*_*j*_ + *x* reduces to *O *(*q*_*j*_) per entry (considering values of the index *h* between *l* and (*k* + 1)*q*_*j*_, and between *j*^′^ and *j*). Entries in matrices of the form *T*^*α*^ and *T*^*ε*^ are computed exactly as done in lines 6 and 7 of Algorithm 1, respectively.

In the second stage, column *j* is computed in matrices *EDT*^*α*^ and *ED*. This is achieved by extending Equations 13 and 12 to have an entire column on the left-hand side, as follows:

(15) $$\begin{array}{l} \;\begin{array}{l} \text{ED}T^{\;\alpha }\left[I_{2,i+1},j\right]\overset{\text{Eq.13}}{\leftarrow }\text{ED}\left[I_{2,i+1},I_{1,j}\right]⊗T^{\alpha }\left[I_{1,j},j\right] \end{array} \end{array}$$

(16) $$\;\begin{array}{l} \text{ED}\left[I_{2,i+1},j\right]\overset{\text{Eq.12}}{\leftarrow }\\ \;\text{min}\left\{\left(\begin{array}{l} S^{\alpha }\left[0,I_{2,i+1}\right]+T^{\alpha }[0,j],\\ \text{tr}(S^{\alpha })\left[I_{2,i+1},I_{1,i+1}\right]⊗\text{ED}T^{\;\alpha }\left[I_{1,i+1},j\right] \end{array}\right|\alpha \in \Sigma \right\} \end{array}$$

This completes the update of the DP tables due to the addition of the letter *t*_*j*-1_.

#### ***Complexity analysis***

After receiving *n* letters, the prefixes *s*_0,*i*_ and *t*_0,*j*_ of the input strings were preprocessed for some *i*,*j* such that *i* + *j* = *n*. The maintenance of lookup tables for efficient *D*-discrete multiplications requires at most $O\left(\frac{|\Sigma |n^{1+\lambda }}{\left|D\right|}\right)$ operations per step among the first *n* steps, and $O\left(\frac{|\Sigma |n^{2+\lambda }}{\left|D\right|}\right)$ operations for all first *n* steps, as shown in Section “Online preprocessing of *D*-discrete matrices”.

Adding a letter *t*_*j*-1_ to the instance, the time required for processing the entries in column *j* of the *T* matrices is as follows. $O\left(\frac{|\Sigma |j}{q_{j}}\right)$ vectors *y*^*α*,*k*^ need to be computed, each vector is computed in $O\left(\frac{j}{q_{j}}\right)$ time, and their total computation time is therefore $O\left(\frac{|\Sigma |j^{2}}{q_{j}^{2}}\right)$. In addition, *O *(|*Σ*|*j*) entries in tables *T*^′*α*^ are computed in *O *(*q*_*j*_) time each, and *O *(|*Σ*|*j*) entries in tables *T*^*α *^and *T*^*ε *^are computed in *O *(|*Σ*|) time each. Therefore, the total time for computing column *j* in all these matrices is $O\left(\frac{|\Sigma |j^{2}}{q_{j}^{2}}+|\Sigma {|}^{2}j\right)=O\left(\frac{|\Sigma |n^{2}}{\lambda^{2}\overset{2}{\underset{\left|D\right|}{\text{log}}}(n)}+|\Sigma {|}^{2}n\right)$.

Computing column *j* in matrices *EDT*^*α*^ and *ED*, the algorithm performs *O *(|*Σ*|) matrix-vector min-plus multiplications (Equations 15 and 16), each taking $O\left(\frac{n^{2}}{\lambda^{2}\overset{2}{\underset{\left|D\right|}{\text{log}}}(n)}\right)$ time using the algorithm in Section “The EDDC algorithm based on efficient *D*-discrete min-plus matrix-vector multiplication”, and computes the entry-wise minimum of |*Σ*|*i*-length vectors (Equation 16) in *O *(|*Σ*|*i*) time. Hence, the total time complexity of computing column *j* is $O\left(\frac{|\Sigma |n^{2}}{\lambda^{2}\overset{2}{\underset{\left|D\right|}{\text{log}}}(n)}+|\Sigma {|}^{2}n\right)$. Symmetrically, this bounds the running time when the *n*-th letter comes from the source string *s*, and so the total running time over all first *n* steps is $O\left(\frac{|\Sigma |n^{3}}{\lambda^{2}\overset{2}{\underset{\left|D\right|}{\text{log}}}(n)}+|\Sigma {|}^{2}n^{2}\right)$. The algorithm requires $O\left(\frac{|\Sigma |n^{2+\lambda }}{\lambda^{2}\overset{2}{\underset{\left|D\right|}{\text{log}}}(n)}\right)$ space for the computed tables.

### Online VMT algorithms

The online algorithm for EDDC presented in the previous section can be generalized for other problems with similar properties. Specifically, VMT problems [11], which utilize min-plus multiplications and for which it can be guaranteed that computed DP matrices are *D*-discrete, can have their algorithms implemented using the same framework as we have presented above. Thus, in contrast to the general case for VMT problems in which it is required that the complete input be available at the beginning of the algorithm’s run, in the *D*-discrete case the input can be obtained in a streaming model. In addition, the asymptotic time complexity in such cases is slightly reduced with respect to the time complexity of the case of min-plus multiplication of general matrices. A concrete example to such a problem is the RNA base-pairing maximization problem [11,15], in which the difference between adjacent entries (in the single DP matrix the algorithm uses) is either 0 or 1. This property was previously exploited by Frid and Gusfield [14] to obtain an $O\left(\frac{n^{3}}{\text{log}n}\right)$ algorithm for the problem. Using the *D*-discrete min-plus multiplication algorithm presented here, this immediately implies an algorithm having the improved time bound of $O\left(\frac{n^{3}}{\overset{2}{\text{log}}n}\right)$. Additional related problems from the domains of RNA folding and Context Free Grammars (CFGs) parsing fall under the VMT framework, and it is likely that *D*-discreteness can be exploited for accelerating the computation of more problems within this family.

## Additional acceleration using run-length encoding

Let *w* be a string. A maximal substring of *w* containing multiple repeats of the same letter is called a *run* in *w*. The *Run Length Encoding* (RLE) of *w* is a representation of the string in which each run is encoded by the corresponding repeating letter *α* and its repeat count *p* (denoted *α*^*p*^). For example, the string *w* = *aabbbaccc *is a concatenation of the four runs *aa*, *bbb*, *a*, and *ccc*, and its RLE is *a*^2 ^*b*^3 ^*a*^1 ^*c*^3^. Denote by $\tilde{w}$ the *compressed form* of *w*, which replaces each run in *w * by a single occurrence of the corresponding letter. When *n* denotes the length of *w*, ñ will denote the length of the compressed form of *w*. The *run index *ĩ of a letter *w*_*i*_ in *w* is the index of the run in which *w*_*i*_ participates. It can be asserted that the compressed form of the substring *w*_*i*,*j*_ of *w* is the substring ${\tilde{w}}_{ĩ,(\tilde{j-1})+1}$ of $\tilde{w}$. In the above example, $\tilde{w}=\text{abac}$, and therefore ñ=4 (while *n* = 9). The run indices of all letters in *w* are given by the sequence [ 0,0,1,1,1,2,3,3,3], and the compressed form of *w*_3,8_ = *bbacc *is ${\tilde{w}}_{\tilde{3},\tilde{7}+1}={\tilde{w}}_{1,4}=\text{bac}$.

Previous works [7-9] showed how RLE can be exploited for improving the efficiency of EDDC algorithms. In these works it was required that the costs of duplications and contractions be less than the costs of all other operations (the requirement was implicit in [9], see discussion in Section “A comparison with previous works”). This requirement is somewhat unnatural for the application of minisatellite map comparison, since it assumes that mutations, which are typically common events, should cost more than the less common events of duplications and contractions. In this section, we adapt a similar RLE-based acceleration to our EDDC algorithm. The application of this acceleration requires the following constraint over cost functions:

### 

**Constraint 1.** *For every α*,*β* ∈ *Σ*, *dup *(*α*) ≤ *dup *(*β*) + *mut *(*β*,*α*) ≤ *ins *(*α*), and *cont *(*α*) ≤ *cont *(*β*) + *mut *(*α*,*β*) ≤ *del *(*α*).

The constraint *dup *(*β*) + *mut *(*β*,*α*) ≤ *ins *(*α*) implies that it never costs more to replace an insertion of some letter *α * into some nonempty string by the duplication of a letter *β* adjacent to the insertion position, and its consecutive mutation to *α*. Thus, we may assume w.l.o.g that optimal edit scripts do not contain insertions (unless applied to empty strings), or in other words, generation of new letters can only be obtained via duplications. Such an assumption is relatively reasonable in the context of minisatellite map comparison, considering the biological mechanisms that describe generative modifications.

The constraint *dup *(*α*) ≤ *dup *(*β*) + *mut *(*β*,*α*) can be intuitively understood by the example of generating a string of the form *α α β *from a string of the form *α β*. Due to the constraint, it would cost the same or less if the string *α α β *is obtained by duplicating the *α* letter in *α β*, rather than by duplicating the *β* letter and mutating its left copy into *α*. Again, such an assumption is relatively reasonable for the minisatellite map application. Symmetric arguments hold with respect to the constraint over contraction and deletion costs.

### 

**Observation 4.** *Let s and w be strings*. *Then, ed *(*s*,*w β β*) ≤ *ed *(*s*,*w β*) + *dup *(*β*)*, and ed *(*s*,*β β w*) ≤ *ed *(*s*,*β w*) + *dup *(*β*) for every *β* ∈ *Σ*.

The correctness of Observation 4 follows from the existence of a script from *s* to *wβ β* whose cost is *ed *(*s*,*w β*) + *dup *(*β*): this script first applies an optimal script to transform *s* into *w β* at cost *ed *(*s*,*w β*), and then duplicates the last *β* in *w β* at cost *dup *(*β*).

### 

**Lemmma 5.** *Let α*,*β be letters and w* ≠ *ε* *a string*. *When Constraint 1 holds, ed *(*α*,*β w*),*ed *(*α*,*w β*) ≥ *ed *(*α*,*w*) + *dup *(*β*),* and ed *(*βw*,*α*),*ed *(*w β*,*α*) ≥ *ed *(*w*,*α*) + *cont *(*β*).

The proof of Lemma 5 appears in Appendix “Proofs to lemmas corresponding to the run-length encoding based EDDC algorithm”.

Next, we show how to reduce the number of expressions that need to be considered in the EDDC recursive equations, in case Constraint 1 applies. For a string *w* of length at least 2, denote by *R *(*w*) ⊆ *P *(*w*) the set of all partitions (*w*^*a*^,*w*^*b*^) of *w* such the last letter in *w*^*a*^ is different from the first letter in *w*^*b*^. For example, for *w* = *aabbbcdddd*,*R *(*w*) = {(*aa*,*bbbcdddd *), (*aabbb*,*cdddd*),(*aabbbc*,*dddd*)}. Observe that |R(w)|=ñ-1.

We start by describing how to improve the computation efficiency of EDDC for cases in which one of the input strings contains a single letter. Denote by *dupcost *(*w*) the cost of the edit script from $\tilde{w}$ to *w* which generates each run *α*^*p*^ in *w* by applying *p* - 1 duplication operations over the corresponding letter *α* in $\tilde{w}$. Similarly, denote by *contcost *(*w*) the cost of the edit script from *w* to $\tilde{w}$ which reduces each run *α *^*p*^ in *w* by applying *p* - 1 contraction operations over *α*. For example, for *w* = *aabbbbaaccc*, *dupcost *(*w*) = 2 *dup *(*a*) + 3 *dup *(*b*) + 2 *dup *(*c*) and *contcost *(*w*) = 2 *cont *(*a*) + 3 *cont *(*b*) + 2 *cont *(*c*). Note that $\text{dupcost}(w)\geq \text{ed}(\tilde{w},w)$, and $\text{contcost}(w)\geq \text{ed}(w,\tilde{w})$. It is simple to assert the following recursive relations: [b]

(17) $$\begin{array}{l} \text{dupcost}(w\beta )=\left\{\begin{array}{ll} \text{dupcost}(w)+\text{dup}(\beta ), & w\;\text{ends with}\;\beta ,\\ \text{dupcost}(w), & \text{otherwise}. \end{array}\right) \end{array}$$

(18) $$\begin{array}{l} \text{dupcost}(w)=\left\{\begin{array}{ll} \text{dupcost}(w^{a})+\text{dupcost}(w^{b}), & (w^{a},w^{b})\in R(w),\\ \text{dupcost}(w^{a}\beta )+\text{dupcost}(\beta w^{b})+\text{dup}(\beta ), & (w^{a}\beta ,\beta w^{b})\in P(w). \end{array}\right) \end{array}$$

The following lemma shows that when one of the input strings contains a single letter, the edit distance can be inferred from the edit distance between this letter and the compressed form of the second string.

### 

**Lemma 6.** *Let α be a letter and w a string*. *When Constraint 1 holds,*$\text{ed}(\alpha ,w)=\text{ed}(\alpha ,\tilde{w})+\text{dupcost}(w)$*, and *$\text{ed}(w,\alpha )=\text{contcost}(w)+\text{ed}(\tilde{w},\alpha )$.

The following lemma shows that given a certain edit script from string *u*, its cost is greater than or equal to the cost of its application on a superstring of *u*.

For a string *s* of the form *s* = *s*^*a *^*us*^*b*^ and an edit script $ES=\langle u=u^{0},u^{1},\ldots ,u^{r}=w\rangle$ from *u* to *w*, denote by ES(s) the edit script $ES(s)=\langle s=s^{a}us^{b}=s^{a}u^{0}s^{b},s^{a}u^{1}s^{b},\ldots ,s^{a}u^{r}s^{b}=s^{a}ws^{b}\rangle$* from s* = *s*^*a*^*us *^*b *^to *t* = *s*^*a*^*ws*^*b*^.

### 

**Lemma 7.** *For s* = *s*^*a*^*us *^*b*^ *and *$ES=\langle u=u^{0},u^{1},\ldots ,u^{r}=w\rangle$, $\text{cost}\left(ES(s)\right)\leq \text{cost}(ES)$.

The proofs of Lemma 6 and Lemma 7 appear in the Appendix.

Equations 17 and 18 and Lemma 6 support the following preprocessing algorithm, Algorithm 3. Given a target string *t*, Algorithm 3 generates data structures that enable retrieving in constant time values of the form *ed *(*α*,*t*_*i*,*j*_) for every *α* ∈ *Σ* and every substring *t*_*i*,*j*_ of *t*. The algorithm generates tables of the form ${\tilde{T}}^{\alpha }$ for every *α* ∈ *Σ*, such that entries ${\tilde{T}}^{\alpha }[\;i,j]$ contain the corresponding values $\text{ed}\left(\alpha ,{\tilde{t}}_{i,j}\right)$. In addition, the algorithm generates a vector *DC*, such that entries *DC *[ *j*] contain the corresponding values *dupcost *(*t*_0,*j*_). Then, queries of the form *ed *(*α*,*t*_*i*,*j*_) can be answered in a constant time according to Equation 19 below.

(19) $$\;\begin{array}{ll} \text{ed}\left(\alpha ,t_{i,j}\right) & \overset{\begin{array}{l} \text{Lem.6}\\ \text{Eq.18} \end{array}}{=}{\tilde{T}}^{\alpha }[\;ĩ,(\tilde{j-1})+1]\\ & \;+\left\{\begin{array}{ll} \text{DC}[\;j]-\text{DC}[\;i], & t_{i-1}\neq t_{i},\\ \text{DC}[\;j]-\text{DC}[\;i]-\text{dup}(t_{i}), & \text{otherwise}. \end{array}\right) \end{array}$$

### **Algorithm 3 RL-LETTER-TO-STRING(*t*)**

An algorithm which is symmetric to Algorithm 3 can be described in order to preprocess a string *s* for queries of the form *ed *(*s*_*i*,*j*_,*α*).

We continue to describe the improved computation in the case where both input strings *s* and *t* are of length at least 2. To do so, we first add some auxiliary notation. For an interval *I*_*x*,*y*_ of positions within a string *w*, denote by ${Ĩ}_{x,y}$ the subsequence of indices in *I*_*x*,*y*_ which are start positions of runs in *w*. For example, for *w* = *aabbbaacccc*, the interval *I*_1,7_ = [ 1,2,…,6] contains all positions of letters within the substring *w*_1,7_ = *abbbaa*, and ${Ĩ}_{1,7}=[\;2,5]$ contains the start positions in *w* of the runs *bbb* and *aa* that are included in *I*_1,7_ (the first letter *a* in *w*_1,7_ belongs to a run in *w* that starts in position 0, and therefore position 1 is not included in ${Ĩ}_{1,7}$). This notation will be used for defining subsequences of rows and columns in DP matrices maintained by the algorithm, where some of these intervals are derived from the source string *s*, and some from the target string *t*. We will assume that the string from which ${Ĩ}_{x,y}$ was derived is clear from the context, and will not specify it explicitly. For example, when ${Ĩ}_{x,y}$ defines rows in matrices *ED* or *EDT*^*α*^, or either rows or columns in matrices *S*^*α*^, then the indices in ${Ĩ}_{x,y}$ are derived from the source string *s*. When ${Ĩ}_{x,y}$ defines columns in matrices *ED* or *EDT*^*α*^, or either rows or columns in matrices *T*^*α*^, then the indices in ${Ĩ}_{x,y}$ are derived from the target string *t*. Subsequences ${Ĩ}_{x,y}$ will be used for defining *sparse regions* in matrices, i.e. regions containing sets of rows or columns which are not necessarily adjacent.

Consider the computation of *ed *(*s*,*t*) as expressed in Equation 8. Assume first the special case where *s* ends with a run of length at least 2. In this case, *s* is of the form *s* = *w β β* for some string *w* and a letter *β*. For every partition (*t*^*a*^,*t*^*b*^) of *t*, it is possible to combine an optimal script ${ES}^{1}$ from the prefix *wβ* of *s* to *t*^*a*^ and an optimal script ${ES}^{2}$ from the suffix *β* of *s* to *t*^*b*^, and to obtain a script $ES=\langle {ES}^{1}(w\beta \beta ),{ES}^{2}(t^{a}\beta )\rangle$ from *s* to *t*. Therefore, $\text{ed}(w\beta \beta ,t)\leq \text{cost}(ES)=\text{cost}({ES}^{1}(w\beta \beta ))+\text{cost}({ES}^{2}(t^{a}\beta ))\overset{\text{Lem.7}}{\leq }\text{cost}({ES}^{1})+\text{cost}({ES}^{2})=\;\text{ed}(w\beta ,t^{a})+\;\text{ed}(\beta ,t^{b})$. In particular, $\text{ed}(w\beta \beta ,t)\leq \text{min}\left\{\text{ed}(w\beta ,t^{a})+\text{ed}(\beta ,t^{b})\;|\;(t^{a},t^{b})\in P(t)\right\}\overset{\text{Eq.9}}{=}\text{ed}t^{\beta }(w\beta ,t)$. In addition, it is possible to compose an edit script from *w β β *to *t* by first contracting the last two letters to obtain the string *wβ*, and then applying an optimal script from *w β* to *t*. The cost of such a script is *ed *(*wβ*,*t*) + *cont *(*β*), and therefore we get that *ed *(*w β β*,*t*)≤ min{*edt*^*β *^(*w β*,*t*),*ed *(*wβ*,*t*) + *cont *(*β*)}.

Next, we show that *ed *(*w β β*,*t*) ≥ min {*edt *^*β *^(*w β*,*t*), *ed *(*w β*,*t*) + *cont *(*β*)}. From Equation 8, either *ed *(*w β β*,*t*) = *ed *(*w β β*,*α*) + *ed *(*α*,*t*) or *ed *(*w β β*,*t*) = *edt *^*α *^(*s*^*a*^,*t*) + *ed *(*s*^*b*^,*α*) for some *α* ∈ *Σ* and (*s*^*a*^,*s*^*b*^) ∈ *P *(*w β β*). Consider first the latter case. If (*s*^*a*^,*s*^*b*^) = (*w β*,*β*), then

$$\;\begin{array}{ll} \text{ed}(w\beta \beta ,t) & =\text{ed}t^{\alpha }(w\beta ,t)+\text{ed}(\beta ,\alpha )\\ & \;\overset{\text{Eq.9}}{=}\text{min}\left\{\text{ed}\left(\mathrm{w\beta},t^{a}\right)+\text{ed}\left(\alpha ,t^{b}\right)\;|\;\left(t^{a},t^{b}\right)\in P(t)\right\}\\ & \;+\text{ed}(\beta ,\alpha )\\ & \;\overset{\text{Obs.5}}{\geq }\text{min}\left\{\text{ed}\left(w\beta ,t^{a}\right)+\text{ed}\left(\beta ,t^{b}\right)\;|\;\left(t^{a},t^{b}\right)\in P(t)\right\}\\ & \;\overset{\text{Eq.9}}{=}\text{ed}t^{\beta }(w\beta ,t). \end{array}$$

Else, *s*^*b*^ is of length at least 2, and there is some string *u* such that *s*^*b *^= *u β β *and *w* = *s*^*a*^*u*. In this case, $\text{ed}(w\beta \beta ,t)=\text{ed}t^{\alpha }\left(s^{a},t\right)+\text{ed}(u\beta \beta ,\alpha )\overset{\text{Lem.5}}{\geq }\text{ed}t^{\alpha }\left(s^{a},t\right)+\text{ed}(u\beta ,\alpha )+\text{cont}(\beta )\overset{\text{Eq.8}}{\geq }\text{ed}(w\beta ,t)+\text{cont}(\beta )$. Similarly, it can be shown that when *ed *(*w β β*,*t*) = *ed *(*w β β*,*α*) + *ed *(*α*,*t*) for some *α *∈ *Σ*, *ed *(*w β β*,*t*) ≥ *ed *(*w β*,*t*) + *cont *(*β*), and so *ed *(*w β β*,*t*) ≥ min {*edt *^*β *^(*w β*,*t*),*ed *(*w β*,*t*) + *cont *(*β*)}. Thus,

(20) $$\text{ed}(w\beta \beta ,t)=\text{min}\left\{\text{ed}t^{\beta }(w\beta ,t),\text{ed}(w\beta ,t)+\text{cont}(\beta )\right\}$$

Formulating Equation 20 with respect to the data structures defined in Section “A baseline dynamic-programming algorithm for EDDC” (under the assumption that all values appearing at the right-hand side of the equation are computed and stored in the corresponding entries), we get the following equation:

(21) $$\begin{array}{ll} \text{ed}\left(s_{0,i},t_{0,j}\right) & =\text{min}\left\{\text{ED}T^{\;s_{i-1}}[\;i-1,j],\text{ED}[\;i-1,j]\right)\\ & \;\left(+\;\text{cont}(s_{i-1})\right\}(\text{when}\;s_{i-1}=s_{i-2}) \end{array}$$

Now, consider the case where the last run in *s* is of length 1 (i.e. *s* is not of the form *w β β*). Assume first that the term that yields the minimum value of the right-hand side of Equation 8 is of the form *edt *^*α*^(*s*^*a*^,*t*) + *ed *(*s*^*b*^,*α*) for some partition (*s*^*a*^,*s*^*b*^) ∈ *P *(*s*) and a letter *α* ∈ *Σ*. If (*s*^*a*^,*s*^*b*^) ∉ *R*(*s*), then there is some letter *β* ∈ *Σ* which is both the last letter of *s*^*a*^ and the first letter of *s*^*b*^. In this case, we can write *s*^*a*^ = *wβ* and *s*^*b*^ = *β u*. Note that *u* ≠ *ε* (since *s* ≠ *w β β* by definition), and so $\text{ed}(s,t)=\text{ed}t^{\alpha }(w\beta ,t)+\text{ed}(\beta u,\alpha )\overset{\text{Eq.9}}{=}\text{min}\left\{\text{ed}\left(w\beta ,t^{a}\right)+\text{ed}\left(\alpha ,t^{b}\right)\;|\;\left(t^{a},t^{b}\right)\in P(t)\right\}+\text{ed}(\beta u,\alpha )\overset{\begin{array}{l} \text{Lem.5},\\ \text{Obs.4} \end{array}}{\geq }\text{min}\left\{\left(\text{ed}\left(w\beta \beta ,t^{a}\right)-\text{dup}(\beta )\right)+\text{ed}\left(\alpha ,t^{b}\right)\;|\;\left(t^{a},t^{b}\right)\in P(t)\right\}+\left(\text{ed}(u,\alpha )+\text{dup}(\beta )\right)=\text{min}\left\{\text{ed}\left(w\beta \beta ,t^{a}\right)+\text{ed}(\alpha ,t^{b})\;|\;\left(t^{a},t^{b}\right)\in P(t)\right\}+\text{ed}(u,\alpha )\overset{\text{Eq.9}}{=}\text{ed}t^{\alpha }(w\beta \beta ,t)+\text{ed}(u,\alpha )$. From the optimality of the partition (*s*^*a*^,*s*^*b*^) = (*wβ*,*βu*), it follows that *ed *(*s*,*t*) = *edt*^*α *^(*w β β*,*t*) + *ed *(*u*,*α*). If *u* starts with *β* this step can be repeated, and inductively we can apply such partition refinements until obtaining a partition (*s*^*a*^,*s*^*b*^) of *s* such that *ed *(*s*,*t*) = *edt*^*α *^(*s*^*a*^,*t*) + *ed *(*s*^*b*^,*α*) and (*s*^*a*^,*s*^*b*^) ∈ *R *(*s*). Now, Equation 8 can be revised as follows:

(22) $$\begin{array}{ll} \text{ed}(s,t) & =\text{min}\left\{\begin{array}{l} \text{ed}(s,\alpha )+\text{ed}(\alpha ,t),\\ \text{ed}t^{\alpha }\left(s^{a},t\right)+\text{ed}\left(s^{b},\alpha \right) \end{array}\left|\begin{array}{l} \left(s^{a},s^{b}\right)\in R(s),\\ \alpha \in \Sigma \end{array}\right)\right\}\\ & \;\;(\text{when}\;s\;\text{is not of the form}\;\mathrm{w\beta \beta}) \end{array}$$

Using the DP formulation, we get

(23) $$\begin{array}{ll} \text{ed}\left(s_{0,i},t_{0,j}\right) & =\text{min}\left\{\begin{array}{l} S^{\alpha }[\;0,i]+T^{\alpha }[\;0,j],\\ \text{ED}T^{\;\alpha }[\;h,j]+S^{\alpha }[\;h,i]\; \end{array}\;\left|\;\begin{array}{l} h\in {Ĩ}_{0,i},\\ \alpha \in \Sigma \end{array}\right)\right\}\\ & =\text{min}\left\{\left(\begin{array}{l} S^{\alpha }[\;0,i]+T^{\alpha }[\;0,j],\\ \text{tr}\left(S^{\alpha }\right)[\;i,{Ĩ}_{0,i}]⊗\text{ED}T^{\;\alpha }\left[{Ĩ}_{0,i},j\right] \end{array}\right|\alpha \in \Sigma \right\}\\ & \;\;(\text{when}\;s_{i-1}\neq s_{i-2}) \end{array}$$

Similarly as shown for the computation of *ed *(*s*,*t*), it is possible to revise the computation of *edt*^*α *^(*s*,*t*) under Constraint 1 and obtain the following equations:

(24) $$\;\text{ed}t^{\alpha }(s,w\beta \beta )=\text{min}\left\{\begin{array}{l} \text{ed}t^{\alpha }(s,w\beta )+\text{dup}(\beta ),\\ \text{ed}(s,w\beta )+\text{mut}(\alpha ,\beta ) \end{array}\right\}$$

(25) $$\;\begin{array}{ll} \text{ed}t^{\alpha }\left(s_{0,i},t_{0,j}\right) & =\text{min}\left\{\begin{array}{l} \text{ED}T^{\;\alpha }[\;i,j-1]+\text{dup}\left(t_{j-1}\right),\\ \text{ED}[\;i,j-1]+\;\text{mut}\left(\alpha ,t_{j-1}\right) \end{array}\right\}\\ & \;\;(\text{when}\;t_{j-1}=t_{j-2}) \end{array}$$

(26) $$\;\begin{array}{ll} \text{ed}t^{\alpha }(s,t)= & \;\text{min}\left\{\text{ed}\left(s,t^{a}\right)+\text{ed}\left(\alpha ,t^{b}\right)\;|\;\left(t^{a},t^{b}\right)\in R(t)\right\}\\ & \;(\text{when}\;t\;\text{is not of the form}\;\mathrm{w\beta \beta}) \end{array}$$

(27) $$\;\begin{array}{ll} \text{ed}t^{\alpha }\left(s_{0,i},t_{0,j}\right) & =\;\text{min}\left\{\text{ED}[\;i,h]+T^{\alpha }[\;h,j]\;|\;h\in {Ĩ}_{0,j}\right\}\\ & =\;\text{ED}\left[\;i,{Ĩ}_{0,j}\right]⊗T^{\alpha }\left[{Ĩ}_{0,j},j\right]\\ & \;\left(\text{when}\;t_{j-1}\neq t_{j-2}\right) \end{array}$$

Finally, we present Algorithm 4, which is an efficient version of Algorithm 2. Stage 1 of the new algorithm is accelerated by using Algorithm 3 to compute for every *α*∈*Σ* distances from *α* all substrings of *s* and distances from all substrings of *t* to *α*. In Stage 2, we use the equations developed above in order to accelerate the computation. The correctness of the algorithm follows from the correctness of the recursive equations, and can be asserted similarly as done for Algorithm 2.

### **Algorithm 4 RL-MATRIX-EDDC(*s*,** ***t*)**

### 

#### ***Complexity analysis***

Assume for simplicity that compressed forms of both input strings *s* and *t* have the same length ñ.

##### 

**Algorithm 3.** The running time of line 1 of the algorithm is *O *(*n*) for computing the compressed form of the input string, and O(|Σ|MP(ñ)) for running Stage 1 of Algorithm 2 over this compressed string. Lines 2 and 3 require *O *(*n*) time, and so the overall time complexity of Algorithm 3 is O(n+|Σ|MP(ñ)). The space complexity for computing and maintaining all matrices is $O(|\Sigma |{ñ}^{2})$, and an additional *O *(*n*) space is required for the vector *DC*. Hence, the overall space complexity of the algorithm is $O(n+|\Sigma |{ñ}^{2})$ (see Section “Time complexity analysis” for complexity analysis of Stage 1 of Algorithm 2).

##### 

**Algorithm 4.** Time and space complexities of Stage 1 of the algorithm are identical to those of Algorithm 3. As in Section “The algorithm”, the computations governing the running time of Stage 2 are those of matrix multiplications performed within recursive calls to RL-COMPUTE-MATRIX.

The recursive computation of RL-COMPUTE-MATRIX can be visualized as a tree (see Figure 4). Each node in the tree corresponds to a call to RL-COMPUTE-MATRIX over some regions *I*_*i*,*k*_ × *I*_*j*,*l*_, which is either a leaf in case that *k* = *i* + 1 and *l* = *j* + 1, or otherwise an internal node. In the latter case, the node has exactly two children, corresponding to the two recursive calls obtained from either a vertical (lines 11 and 13) or a horizontal (lines 16 and 18) partition of the region. For simplicity, assume that the interval length $ñ=\left|{Ĩ}_{2,n+1}\right|=2^{x}$ for some integer *x*. It can be observed that the algorithm alternates between vertical and horizontal partitions along paths from the root of the tree, where regions of two different nodes in the same depth *y* are disjoint, and the union of all regions of nodes in depth *y* covers the entire initial region *I*_2,*n* + 1_ × *I*_2,*n* + 1_ of the root node. For every 0 ≤ *y* ≤ log(*n*), there are two series of intervals $I^{y,0},I^{y,1},\ldots ,I^{y,2^{y}-1}$ and $J^{y,0},J^{y,1},\ldots ,J^{y,2^{y}-1}$, such that the set of regions corresponding to all nodes in depth 2*y* is {*I*^*y*,*f *^× *J*^*y*,*g*^ | 0 ≤ *f * < 2^*y*^,0 ≤ *g* < 2^*y*^}, and the set of regions corresponding to all nodes in depth 2 *y* + 1 is {*I*^*y*,*f *^× *J*^*y* + 1,*g*^ | 0 ≤ *f* < 2^*y*^,0 ≤ *g* < 2^*y* + 1^}. In addition, the corresponding subsequences ${Ĩ}^{y,0},\ldots ,{Ĩ}^{y,2^{y}-1}$ and ${\tilde{J}}^{y,0},\ldots ,{\tilde{J}}^{y,2^{y}-1}$ have all the same size 2^*x* - *y*^.

Consider a node of depth 2*y* whose corresponding region is *I*^*y*,*f*^ × *J*^*y*,*g*^, and the two regions corresponding to its children *I*^*y*,*f*^ × *J*^*y* + 1,2*g*^ and *I*^*y*,*f*^ × *J*^*y* + 1,2*g* + 1^. The computation time spent on the node is dominated by the matrix multiplications performed in line 12 of RL-COMPUTE-MATRIX. This includes |*Σ*| matrix multiplications between pairs of matrices such the dimensions of the first matrix in each pair is $\left|I^{y,f}\right|\times \left|{\tilde{J}}^{y+1,2g}\right|=\left|I^{y,f}\right|\times 2^{x-y-1}$, and the dimensions of the second matrix in a pair is $\left|{\tilde{J}}^{y+1,2g}\right|\times \left|{\tilde{J}}^{y+1,2g+1}\right|=2^{x-y-1}\times 2^{x-y-1}$. Observe that |*I*^*y*,*f *^|≥ 2^*x*-*y*-1^, and such multiplications can be implemented by dividing the interval *I*^*y*,*f*^ into $\frac{\left|I^{y,f}\right|}{2^{x-y-1}}$ intervals of length 2^*x*-*y*-1^ each, and performing $\frac{\left|I^{y,f}\right|}{2^{x-y-1}}$ multiplications between square matrices of dimensions 2^*x*-*y*-1^ × 2^*x*-*y*-1^ in a total time of $\frac{\left|I^{y,f}\right|}{2^{x-y-1}}\text{MP}(2^{x-y-1})$. Therefore, the time required for all matrix multiplications performed within nodes in depth 2 *y* is

$$\begin{array}{ll} \underset{0\leq f<2^{y}}{\sum }\underset{0\leq g<2^{y}}{\sum } & \frac{\left|\Sigma |\left|I^{y,f}\right|\text{MP}(2^{x-y-1}\right)}{2^{x-y-1}}\\ & \;=\underset{0\leq f<2^{y}}{\sum }\frac{2^{y}|\Sigma |\left|I^{y,f}\right|\text{MP}(2^{x-y-1})}{2^{x-y-1}}\\ & \;=\frac{2|\Sigma |\text{nMP}(2^{x-y-1})}{ñ}. \end{array}$$

Similarly, it is possible to show that the total time required for all matrix multiplications performed within nodes in depth 2 *y* + 1 is also $\frac{2n|\Sigma |\text{MP}(2^{x-y-1})}{ñ}$, and so the total computation time of matrix multiplications throughout the entire algorithm run is $O\left(\frac{|\Sigma |n}{ñ}\underset{0\leq y<x}{\sum }\text{MP}(2^{y})\right)$. As in Section “The algorithm”, using the Master Theorem [16], this summation evaluates to $O\left(\frac{\left|\Sigma |\text{nMP}(ñ\right)}{ñ}\right)$. In addition to matrix multiplications, RL-COMPUTE-MATRIX performs *O *(|*Σ*|*n*^2^) operations in base computations (lines 2-7), and so the total time complexity of the complete algorithm is $O\left(|\Sigma |n^{2}+\frac{\left|\Sigma |\text{nMP}(ñ\right)}{ñ}\right)$.

A simple implementation of Algorithm 4 can be done using the same space complexity of *O *(|*Σ*|*n*^2^), as the space complexity of Algorithm 2. A more involved implementation can be applied by observing that in fact the algorithm only examines and updates entries in matrices of dimensions at most n×ñ or ñ×n when performing matrix multiplications, and in addition it examines adjacent entries “to the left” or “above” an entry in a base-case region. This observation can be used in order to reduce the space complexity of the algorithm to $O\left(|\Sigma |\mathrm{nñ}\right)$, where the complete details of such an implementation are omitted from this text.

## A comparison with previous works

In this section, we review the previous main algorithms for EDDC by Behzadi and Steyaert [7], Bérard et al. [8] and Abouelhoda et al. [9], and point out similarities and improvements made in our current work.

The main contribution of our work is in obtaining sub-cubic algorithms for EDDC, whereas all previous algorithms have cubic time complexities (for |*Σ*| the alphabet size, *n* the length of the input strings and ñ the length of their RLE compressed forms, the algorithms of [7], [8], and [9] obtain the time complexities $O\left(n^{2}+n{ñ}^{2}+|\Sigma |{ñ}^{3}+|\Sigma {|}^{2}{ñ}^{2}\right)$, $O\left(n^{3}+|\Sigma |{ñ}^{3}\right)$, and $O(n^{2}+n{ñ}^{2})$, respectively).

Notably, the algorithm of [9] eliminates a |*Σ*| factor that appears in the time complexities of the algorithms given in [7,8] and here. However, this improvement is confined to a constrained model of duplication histories. As we do not assume this model here, we could not use the representation of [9] that allows the elimination of the |*Σ*| time complexity factor.

In general, the frameworks of all algorithms in [7-9] as well as the algorithms presented here are similar. All these algorithms apply two phases, where the first phase computes costs corresponding to all substrings of each one of the input strings separately, and the second phase uses these precomputed costs in order to compute the edit distance between each pair of prefixes of the input strings (our online variant described in Section “An online algorithm for EDDC using min-plus matrix-vector multiplication for discrete cost functions” interleaves these two phases, yet each operation it conducts can be conceptually attributed to one of the phases). The recursive formulas are similar as well, where those for the first phase can be viewed as special kinds of Weighted Context Free Grammar derivation rules.

Next, we address the cost function constraints. All algorithms assume that operation costs are nonnegative and apply additional assumptions similarly to those listed in our Property 1, which can be made without loss of generality.

In [8], operation costs were limited so that all duplications and contractions have the same constant cost (regardless of the letter over which they are applied), all deletions and insertions have the same constant cost, and all mutation costs are symmetric (i.e. *mut *(*α*,*β*) = *mut *(*β*,*α*) for every *α*,*β* ∈ *Σ*). While it was argued that these restrictions allow edit distance to be a metric, they limit the generality of the algorithm of [8], where the rest of the previous algorithms we mentioned can handle scoring schemes that not necessarily abide by these restrictions.

Both in [7] and in [8], it was required that all duplication and contraction costs are lower than the costs of any of the insertion, deletion, or mutation costs. This restriction is not explicitly stated in [9], yet seems to be required there as well. For the application of minisatellite map comparison, this requirement is somewhat unnatural since it assumes that mutations, which are typically common events, should cost more than the less common events of duplications and contractions. Our algorithms can be applied even when this restriction does not hold. However, one of our algorithms, the RLE variant (Section “Additional acceleration using run-length encoding”) adds a new requirement that was absent from those previous algorithms: it requires that for every *α*,*β* ∈ *Σ*, *dup *(*α*) ≤ *dup *(*β*) + *mut *(*β*,*α*) ≤ *ins *(*α*), and *cont *(*α*) ≤ *cont *(*β*) + *mut *(*α*,*β*) ≤ *del *(*α*) (our Constraint 1). On one hand, our Constraint 1 is more strict than the constraint of [7] and [8], in the sense that it implies nonnegative lower bounds over differences of the form *ins *(*α*) - *dup *(*α*) and *del *(*α*) - *cont *(*α*), while in [7] and [8] it was only required that these differences be nonnegative. On the other hand, our Constraint 1 does not require that the cost of mutations be higher than the cost of duplications and contractions.

We showed that our algorithms are more general with respect to the assumed constraints. We also claim that our algorithms are more precise with respect to the formal problem specification. All previous algorithms (excluding the first algorithm by Bérard and Rivals [2], which had an *O *(*n*^4^) running time and assumed a constant cost for all mutations in addition to the restrictions in [8]) might output non-optimal solutions in certain cases, as demonstrated in the following example. Consider the input *s* = *ab*, *t* = *ef*, and the cost function in which all duplications and contractions cost 1, all deletions and insertions cost 20, and the symmetric mutation costs are as given in Table 1. It can be shown that all three algorithms in [7], [8], and [9] would output the value 18 as the edit distance between the input strings, reflecting one of the edit scripts 〈*ab*,*eb*,*ef*〉 or 〈*ab*,*af*,*ef*〉. Nevertheless, the correct value is 17, due to the script 〈*ab*,*cb*,*cc*,*c*,*d*,*dd*,*ed*,*ef*〉. Perhaps it could be possible to specify additional restrictions over the cost functions in order to guarantee that the algorithms in [7], [8], and [9] return optimal solutions for all instances.

**Table 1:** **Mutation costs for the instance *s * = *ab *, *t * = *ef***

	**a**	**b**	**c**	**d**	**e**	**f**
**a**	0	6	3	6	9	9
**b**	6	0	3	6	9	9
**c**	3	3	0	3	6	6
**d**	6	6	3	0	3	3
**e**	9	9	6	3	0	6
**f**	9	9	6	3	6	0

## Conclusions and discussion

This work presents computational techniques for improving the time complexity of algorithms for the EDDC problem. We adapt the problem to the *VMT* framework defined in [11], which incorporates efficient matrix multiplication subroutines in order to accelerate standard dynamic programming algorithms. We describe an efficient algorithm, as well as two variants which are even more efficient, given some restrictions on the cost functions.

An additional result we give is the currently most efficient algorithm for the min-plus multiplication of *D*-discrete matrices (matrices for which differences between adjacent entries are integers within an interval of length *D*).

We note that the running times of our algorithms depend on the alphabet size |*Σ*|. For the general algorithm, the running time is *O *(|*Σ*|·*MP*(*n*)), where *MP*(*n*) is the time complexity of the min-plus multiplication of two *n* × *n* matrices, which is currently upper-bounded by $O\left(\frac{n^{3}\overset{3}{\text{log}}\text{log}n}{\overset{2}{\text{log}}n}\right)$[12]. Some of the previous algorithms obtain alphabet independent time complexities, for example the algorithms in [9] and [2]. As we discussed in Section “A comparison with previous works”, such algorithms do not solve the most general variant of the problem and require some assumptions on the cost function. Nevertheless, we believe that the matrix multiplication-based techniques for improving the time complexity presented in this paper can also be incorporated to the algorithm of [9], however the details of this enhancement are beyond the scope of this paper.

In contrast to the work of [9], our model assumes that intermediate strings along edit scripts may contain characters which are absent from both source and target strings. This implies that the size of the alphabet |*Σ*| is not bounded by the length of the input sequences. In the context of minisatellite comparison, identifying a feasible alphabet and cost function for this task is an interesting problem beyond the scope of this paper.

## Appendix

### Correctness of the recursive computation

This section proves Theorem 1, thus asserting the correctness of the recursive computation for the EDDC problem given in Section “The recurrence formula”. We start by adding some required notation and showing how long edit scripts can be decomposed to shorter partial scripts. Then, we use the observed recursive properties in order to prove the correctness of the recurrence.

Let *s*,*w*,*t* be strings, ${ES}^{1}=\langle s=u^{1,0},u^{1,1},\ldots ,u^{1,r_{1}}=w\rangle$ an edit script from *s* to *w*, and ${ES}^{2}=\langle w=u^{2,0},u^{2,1},\ldots ,u^{2,r_{2}}=t\rangle$ an edit script from *w* to *t*. Denote by $ES=\langle {ES}^{0},{ES}^{1}\rangle$ the concatenated edit script $ES=\langle s\;=\;u^{1,0},u^{1,1},\ldots ,u^{1,r_{1}}=w=u^{2,0},u^{2,1},\ldots ,u^{2,r_{2}}=t\rangle$ from *s* to *t*. Note that $\text{cost}(ES)=\text{cost}\left({ES}^{1}\right)+\text{cost}\left({ES}^{2}\right)$, and $\left|ES\right|=\left|{ES}^{1}\right|+\left|{ES}^{2}\right|$. This notation extends naturally to concatenations of more than two scripts. For example, $ES=\langle {ES}^{1},{ES}^{2},\ldots ,{ES}^{q}\rangle$ denotes an edit script from a string *s* to a string *t* obtained by a concatenation of *q* scripts, each script ${ES}^{i}$ transforms some intermediate string *w*^*i*-1^ into a string *w*^*i*^, and *s* = *w*^0^ and *t* = *w*^*q*^.

#### 

**Obeservation 5.** *For every three strings s*,*w*,*t*, *ed*(*s*,*t*) ≤ *ed *(*s*,*w*) + *ed *(*w*,*t*).

The correctness of the above observation follows from the fact that for a pair of optimal edit scripts ${ES}^{1}$ from *s* to *w* and ${ES}^{2}$ from *w* to *t*, the script $ES=\langle {ES}^{1},{ES}^{2}\rangle$ from *s* to *t* satisfies $\text{ed}(s,t)\leq \text{cost}(ES)=\text{cost}({ES}^{1})+\text{cost}({ES}^{2})=\text{ed}(s,w)+\text{ed}(w,t)$.

Lemma 7.

*Fors* = *s*^*a *^*us *^*b *^*and *$ES=\langle u=u^{0},u^{1},\ldots ,u^{r}=w\rangle$, $\text{cost}\left(ES(s)\right)\leq \text{cost}(ES)$*.*

#### 

*Proof.* Each edit operation transforming *s*^*a *^*u*^*i *^*s*^*b *^to *s*^*a *^*u*^*i *+ 1 ^*s*^*b *^in ES(s) corresponds to an operation transforming *u*^*i *^to *u *^*i* + 1^ in ES. The only cases where corresponding operations may have different costs are those of insertions or deletions in ES at the beginning or ending of *u*^*i*^, which become duplications or contractions in ES(s), respectively. For example, in case the applied operation over *u*^*i *^ in ES is the deletion of its first letter *α*, and *α* is also the last letter of *s*^*a*^, then the cost of the operation in ES is *del *(*α*) while the cost of the corresponding operation in ES(s) is *cont *(*α*) ≤ *del *(*α*). Similar scenarios may occur in case of an insertion of a letter at the beginning of *u*^*i *^which is identical to the last letter of *s*^*a*^, as well as in cases of insertions and deletions at the end of *u*^*i *^of letters identical to the first letter of *s*^*b*^. In any other case, each pair of corresponding operations have the same cost. Therefore the cost of each operation in ES(s) is smaller than or equals to the cost of its corresponding operation in ES, and $\text{cost}\left(ES(s)\right)\leq \text{cost}(ES)$. □

#### 

**Lemma 8.** *Let s and t be two strings, and *(*s*^*a*^, *s*^*b*^) ∈ *P *(*s*), (*t*^*a*^, *t*^*b*^) ∈ *P *(*t*)* partitions of s and t, respectively*. *Then, ed *(*s*,*t*) ≤ *ed *(*s*^*a*^,*t*^*a*^) + *ed *(*s*^*b*^,*t*^*b*^).

#### 

*Proof.* Let ${ES}^{a}$ be an optimal script from *s*^*a*^ to *t*^*a*^ and ${ES}^{b}$ an optimal script from *s*^*b*^ to *t*^*b*^. The script ${ES}^{a}(s)$ is a script from *s* = *s*^*a *^*s*^*b *^to *t*^*a *^*s*^*b*^. Similarly, ${ES}^{b}(t^{a}s^{b})$ is a script from *t*^*a *^*s*^*b *^to *t*^*a *^*t*^*b *^= *t*. For the script $ES=\langle {ES}^{a}(s),{ES}^{b}(t^{a}s^{b})\rangle$ from *s* to *t*, we have that $\text{ed}(s,t)\leq \text{cost}(ES)=\text{cost}({ES}^{a}(s))+\text{cost}({ES}^{b}(t^{a}s^{b}))\overset{\text{Lem.7}}{\leq }\text{cost}({ES}^{a})+\text{cost}({ES}^{b})=\text{ed}\left(s^{a},t^{a}\right)+\text{ed}\left(s^{b},t^{b}\right)$. □

Let *s* and *t* be strings. Call a pair of partitions (*s*^*a*^, *s*^*b*^) ∈ *P *(*s*) and (*t*^*a*^, *t*^*b*^) ∈ *P *(*t*) an *optimal pairwise partition* of *s* and *t* if *ed *(*s*,*t*) = *e *(*s*^*a*^, *t*^*a*^) + *ed *(*s*^*b*^, *t*^*b*^). Say that an edit script ES from *s* to *t* is a *shortest optimal* script from *s* to *t* if ES is optimal, and for every other optimal script ${ES}^{\prime }$ from *s* to *t*, $\left|ES\right|\leq \left|{ES}^{\prime }\right|$. For a script $ES=\langle s=u^{0},u^{1},\ldots ,u^{r}=t\rangle$ from *s* to *t* and 0 ≤ *i* ≤ *j* ≤ *r*, denote by ${ES}^{i,j}=\langle u^{i},u^{i+1},\ldots ,u^{j}\rangle$ the partial script of ES from *u*^*i*^ to *u*^*j*^.

#### 

**Observation 6.** *Let *$ES=\langle u^{0},u^{1},\ldots ,u^{r}\rangle$*be a shortest optimal edit script from u*^0^ *to u*^*r*^. *For every *0 ≤ *i* ≤ *j* ≤ *r, the partial script *${ES}^{i,j}$*is a shortest optimal edit script from u*^*i *^*to u*^*j*^. *Moreover, for any shortest optimal script *${ES}^{*i,j}$*from u*^*i *^*to u*^*j *^*within *ES*, the script *${ES}^{*}=\langle {ES}^{0,i},{ES}^{*i,j},{ES}^{j,r}\rangle$*is a shortest optimal script from u*^0^ to *u*^*r*^.

The correctness of the above observation is obtained by noting that if ${ES}^{i,j}$ is not a shortest optimal script from *u*^*i*^ to *u*^*j*^, then for some shortest optimal script ${ES}^{*i,j}$ from *u*^*i*^ to *u*^*j*^ we get that the script ${ES}^{*}=\langle {ES}^{0,i},{ES}^{*i,j},{ES}^{j,r}\rangle$ either has a lower cost than ES, or is a shorter script of the same cost, in contradiction to ES being a shortest optimal script from *u*^0^ to *u*^*r*^.

#### 

**Lemma 9.** *Let *$ES=\langle u^{0},u^{1},\ldots ,u^{r}\rangle$*be a shortest optimal edit script from u*^0^ to *u*^*r*^. *If there are two indices *0 ≤ *i* < *j* ≤ *r* such that *u*^*i *^*and u*^*j *^*are strings of length 1, then j* = *i* + 1. *In addition, for every *0 < *k* < *r*, *u*^*k *^≠ *ε*.

#### 

*Proof.* Assume there are two indices 0 ≤ *i* < *j* ≤ *r* such that *u*^*i *^and *u*^*j *^are strings of length 1, i.e *u*^*i *^= *α* and *u*^*j *^= *β* for some *α*,*β* ∈ *Σ*. From Observation 6, the partial script ${ES}^{i,j}=\langle \alpha =u^{i},u^{i+1},\ldots ,u^{j}=\beta \rangle$ is a shortest optimal script from *α* to *β*. Since *j* > *i*, it must be that *α* ≠ *β* (otherwise the script ${ES}^{\prime }=\langle {ES}^{0,i},{ES}^{j,r}\rangle$ is a shorter script from *u*^0^ to *u*^*r*^ of no greater cost than ES, in contradiction to ES being a shortest optimal script from *u*^0^ to *u*^*r*^). From Property 1, *ed *(*α*,*β*) = *mut *(*α*,*β*), and so the edit script containing the single operation of mutating *α* to *β* is an optimal script from *α* to *β*, and it must be that *j* = *i* + 1.

In addition, assume by contradiction there is some index 0 < *k* < *r* such that *u*^*k*^ = *ε*. The only edit operation which may yield an empty string is a deletion from a single-letter string, and therefore *u*^*k*-1^ = *α* for some letter *α*. Similarly, the only edit operation which may be applied over an empty string is an insertion, therefore *u*^*k*+1^ = *β* for some letter *β*, in contradiction to the fact that two intermediate strings of length 1 must be consecutive along a shortest optimal script, as shown above. □

Call an edit script ES from a string *s* to a string *t simple* if ES is a shortest optimal script from *s* to *t*, in which no generating operation precedes a reducing operation. The following lemma generalizes Lemma 2 of [6], by considering also indels in addition to contractions and duplications.

#### 

**Lemma 10.** *For every pair of strings s and t, there exists a simple edit script from s to t*.

#### 

*Proof.* Let *s* and *t* be two strings, and *r* the length of a shortest optimal script from *s* to *t*. When *r* ≤ 1, any shortest optimal script from *s* to *t* either contains no reducing operation or contains no generating operation, and in particular is a simple script. Otherwise, *r* > 1, and assume by induction the lemma holds for every pair of strings such that the length of a shortest optimal script from the source string to the target string is less than *r*. Let $ES=\langle s=u^{0},u^{1},\ldots ,u^{r}=t\rangle$ be a shortest optimal script from *s* to *t*.

**Case 1: The first operation in**ES** is not a generating operation.** From Observation 6, the partial script ${ES}^{1,r}$ is a shortest optimal script from *u*^1^ to *u*^*r*^, whose length is *r* - 1. From the inductive assumption, there is a simple script ${ES}^{*1,r}$ from *u*^1^ to *u*^*r*^, and from Observation 6 the script ${ES}^{*}=\langle {ES}^{0,1},{ES}^{*1,r}\rangle$ is a shortest optimal script from *s* to *t*. As the first operation in ${ES}^{*}$ is non-generating (being the same first operation as in ES), ${ES}^{*}$ is simple, and the lemma follows.

**Case 2: The first operation in**ES** is a generating operation.** Similarly as above, we may assume w.l.o.g. by applying the inductive assumption that the partial script ${ES}^{1,r}$ is simple. If this partial script is non-reducing, then ES is non-reducing, and in particular it is simple. Otherwise, let 1≤*i*<*r* be the smallest index such that the transformation of *u*^*i*^ to *u*^*i*+1^ is by a reducing operation. Since neither generating nor reducing operations may precede this operation in the partial script ${ES}^{1,i}$, it follows that all operations in the partial script ${ES}^{1,i}$ (if there are any) are mutations.

The generating operation transforming *u*^0^ to *u*^1^ is either an insertion or a duplication of some letter *α* in *u*^0^. In both cases, we can write *s* = *u*^0^ = *vxw *and *u*^1^ = *vx*^′^*w* (*v*,*x*,*x*^′^ and *w* are strings), where in the former case *x* = *ε* and *x*^′^ = *α*, and in the latter case *x*=*α* and *x*^′^ = *αα*. As all operations in the partial script ${ES}^{1,i}$ are mutations, each intermediate string *u*^*j*^, for 1 ≤ *j* ≤ *i*, is of the form *v*^*j *^*x*^*j *^*w*^*j*^, where *v*^*j*^,*x*^*j*^, and *w*^*j *^are string obtained by applying zero or more mutations over *v*,*x*^′^, and *w*, respectively. We argue that the reducing operation transforming *u*^*i *^= *v*^*i *^*x*^*i *^*w*^*i *^to *u*^*i*+1^ cannot be the deletion of a letter or a contraction involving at least one letter within the substring *x*^*i*^. This is true, since in such a case it would have been possible to avoid the first generating operation in ES (transforming *x* to *x*^′^), as well as all mutation operations over a reduced letter in *x*^*i*^, and the reducing operation from *u*^*i*^ to *u*^*i*+1^. This would yield a script ${ES}^{*0,i+1}$ from *u*^0^ to *u*^*i*+1^ which is shorter and of no higher coast than ${ES}^{0,i+1}$, in contradiction to Observation 6. Hence, the reducing operation from *u*^*i*^ to *u*^*i*+1^ either deletes a letter or contracts two letters within one of the substrings *v*^*i *^or *w*^*i *^of *u*^*i*^.

Consider first the case where the reducing operation over *u*^*i *^is applied within its prefix *v*^*i*^. Thus, we can write *u*^*i*+1^ = *v*^*i*+1 ^*x*^*i*+1 ^*w*^*i*+1^, where *v*^*i*+1^ is the string obtained by applying the corresponding reducing operation over *v*^*i*^, *x*^*i*+1^ = *x*^*i*^, *w*^*i*+1^ = *w*^*i*^, and *cost *(〈 *u*^*i*^,*u*^*i*+1^ 〉) = *cost *(〈 *v*^*i*^,*v*^*i*+1^ 〉). The operations in ${ES}^{0,i+1}$ can be assigned into two independent scripts: a script ${ES}_{v}=\langle v=v^{\prime 0},v^{\prime 1},\ldots ,v^{\mathrm{\prime p}}=v^{i+1}\rangle$ from *v* to *v*^*i*+1^ obtained by merging each multiple occurrence of consecutive identical strings in the series *v* = *v*^1^,*v*^2^,…,*v*^*i*+1^ into a single occurrence, and similarly a script ${ES}_{\text{xw}}=\langle \text{xw}={\left(\text{xw}\right)}^{0},{\left(\text{xw}\right)}^{1}=x^{\prime }w=x^{1}w^{1},{\left(\text{xw}\right)}^{2},\ldots ,{\left(\text{xw}\right)}^{q})\;=\;x^{i+1}w^{i+1}(\rangle$ from *xw* to *x*^*i*+1 ^*w*^*i*+1^. Each operation in ${ES}^{0,i+1}$ corresponds to exactly one operation in either ${ES}_{v}$ or ${ES}_{\text{xw}}$, where the costs of corresponding operations are equal, and therefore $\text{cost}({ES}^{0,i+1})=\text{cost}({ES}_{v})+\text{cost}({ES}_{\text{xw}})$ and $\left|{ES}^{0,i+1}|=|{ES}_{v}|+|{ES}_{\text{xw}}\right|$.

Now, the script ${ES}_{v}(u^{0})=\langle u^{0}=\text{vxw}=v^{\prime 0}\text{xw},v^{\prime 1}\text{xw},\ldots ,v^{\prime p}\text{xw}=v^{i+1}\text{xw}\rangle$ is a script from *u*^0^ to *v*^*i*+1 ^*xw*, and similarly the script ${ES}_{\text{xw}}(v^{i+1}\text{xw})=\langle v^{i+1}\text{xw}=v^{i+1}{\left(\text{xw}\right)}^{0},v^{i+1}{\left(\text{xw}\right)}^{1},\ldots ,v^{i+1}{\left(\text{xw}\right)}^{q}=v^{i+1}x^{i+1}w^{i+1}=u^{i+1}\rangle$ is a script from *v*^*i*+1 ^*xw *to *u*^*i*+1^. Thus, the script ${ES}^{*0,i+1}=\langle {ES}_{v}(u^{0}),{ES}_{\text{xw}}(v^{i+1}\text{xw})\rangle$ is a script from *u*^0^ to *u*^*i*+1^. Since ${ES}_{v}$ contains at least one operation (the reducing operation from *v*^*i*^ to *v*^*i*+1^) and no generating operation (since besides the reducing operation ${ES}_{v}$ may contain only mutations), ${ES}^{*0,i+1}$ starts with a non-generating operation. In addition, $\text{cost}({ES}^{*0,i+1})=\text{cost}({ES}_{v}(u^{0}))+\text{cost}({ES}_{\text{xw}}(v^{i+1}\text{xw}))\overset{\text{Lem.7}}{\leq }\text{cost}({ES}_{v})+\text{cost}({ES}_{\text{xw}})=\text{cost}({ES}^{0,i+1})$ and $\left|{ES}^{*0,i+1}\right|=\left|{ES}_{v}(u^{0})\right|+\left|{ES}_{\text{xw}}(v^{i+1}\text{xw})\right|=\left|{ES}_{v}\right|+\left|{ES}_{\text{xw}}\right|=\left|{ES}^{0,i+1}\right|$. From Observation 6, ${ES}^{0,i+1}$ is a shortest optimal script from *u*^0^ to *u*^*i*+1^, and so ${ES}^{*0,i+1}$ is a shortest optimal script from *u*^0^ to *u*^*i*+1^. Applying Observation 6 again, the script ${ES}^{*}=\langle {ES}^{*0,i+1}{ES}^{i+1,r}\rangle$ is a shortest optimal script from *s* to *t*. Now, the lemma follows from Case 1 of this proof and from the fact the first operation in ${ES}^{*}$ is not a generating operation. □

#### 

**Lemma 11.** *For every α* ∈ *Σ and every nonempty string t, any simple script from α to t is non-reducing*.

#### 

*Proof.* Let ES be a simple script from *α* to *t*, and assume by contradiction ES contains a reducing operation. Since ES is simple, all reducing operations in ES occur prior to any generating operation, and in particular the first reducing operation is applied after applying zero or more mutations over *α*. Such a reducing operation must be a deletion from a string of length 1, resulting with an empty intermediate string, in contradiction to Lemma 9. □

#### 

**Lemma 12.** *Let w and t be strings and β a letter, such that w ≠ ε*, *t is of length at least 2, and there is a non-reducing simple script from w β* *to t*. *Then, ed *(*w β*,*t*) *= min *{*ed *(*w*,*t*^*a*^) + *ed *(*β*,*t*^*b*^) | (*t*^*a*^,*t*^*b*^) ∈ *P *(*t*)}.

#### 

*Proof.* Let $ES=\langle \mathrm{w\beta}=u^{0},u^{1},\ldots ,u^{r}=t\rangle$ be a non-reducing simple script from *wβ* to *t*. For every 0 ≤ *i* ≤ *r*, construct a partition (*u*^*i*,*a*^, *u*^*i*, *b*^) of *u*^*i *^which sustains that *ed *(*wβ*, *u*^*i*^) ≥ *ed *(*w*, *u*^*i*, *a*^) + *ed *(*β*,*u*^*i*,*b*^), as follows. For *i* = 0, set (*u*^0,*a*^,*u*^0,*b*^) = (*w*,*β*), where by definition *ed *(*wβ*,*u*^0^) = *ed *(*w*,*u*^0,*a*^) + *ed *(*β*,*u*^0,*b*^) = 0. Now, assume inductively for some 0 < *i* ≤ *r* and a partition (*u*^*i*-1,*a*^, *u*^*i*-1, *b*^) of *u*^*i*-1^ that *ed *(*wβ*, *u*^*i*-1^) ≥ *ed *(*w*, *u*^*i*-1, *a*^) + *ed *(*β*, *u*^*i*-1, *b*^). If the non-reducing operation transforming *u*^*i*-1^ to *u*^*i*^ is a mutation, an insertion, or a duplication of a letter within the prefix *u*^*i*-1, *a*^, then set *u*^*i*,*a*^ to be the string obtained by applying this operation over *u*^*i*-1, *a*^, and set *u*^*i*, *b*^ = *u*^*i*-1, *b*^. Otherwise, the operation is a mutation, an insertion, or a duplication of a letter within the suffix *u*^*i*-1, *b*^, and in this case set *u*^*i*,*b*^ to be the string obtained by applying this operation over *u*^*i*-1, *b*^, and *u*^*i*,*a*^ = *u*^*i*-1, *a*^. Note that in both cases, $\text{cost}({ES}^{i-1,i})=\text{ed}\left(u^{i-1,a},u^{i,a}\right)+\text{ed}\left(u^{i-1,b},u^{i,b}\right)$, therefore we get from the inductive assumption that $\text{ed}\left(w\beta ,u^{i}\right)\overset{\text{Obs.6}}{=}\text{cost}({ES}^{0,i})=\text{cost}({ES}^{0,i-1})+\text{cost}({ES}^{i-1,i})\;\geq \;\left(\text{ed}\left(w,u^{i-1,a}\right)\;+\;\text{ed}\left(\beta ,u^{i-1,b}\right)\right)\;+\;\left(\text{ed}\left(u^{i-1,a},u^{i,a}\right)\;+\;\text{ed}\left(u^{i-1,b},u^{i,b}\right)\right)\;\overset{\text{Obs.5}}{\geq }\;\text{ed}\left(w,u^{i,a}\right)+\text{ed}\left(\beta ,u^{i,b}\right)$.

The process above generates a partition (*t*^∗*a*^, *t*^∗*b*^) = (*u*^*r*, *a*^,*u*^*r*, *b*^) of *t*=*u*^*r*^, for which *ed *(*u*^*i*^, *t*) ≥ *ed *(*w*, *t*^∗*a*^) + *ed *(*β*,*t*^∗*b*^). In particular, *ed *(*wβ*,*t*) ≥ min {*ed *(*w*,*t*^*a*^) + *ed *(*β*,*t*^*b*^) | (*t*^*a*^,*t*^*b*^) ∈ *P *(*t*)}. On the other hand, $\text{ed}(w\beta ,t)\overset{\text{Lem.8}}{\leq }\text{min}\left\{\text{ed}\left(w,t^{a}\right)\;+\;\text{ed}\left(\beta ,t^{b}\right)\;|\;\left(t^{a},t^{b}\right)\;\in \;P(t)\right\}$, and the lemma follows. □

Based on the above observations and lemmas, we now turn to prove the recursive computation given in Section “The recurrence formula”, starting with Equation 7. Fix henceforth a pair of input strings *s* and *t*, each containing at least two letters. Note that for every *α* ∈ *Σ* and every partitions (*s*^*a*^, *s*^*b*^) ∈ *P *(*s*) and (*t*^*a*^, *t*^*b*^) ∈ *P *(*t*), *ed *(*s*,*t*) ≤ Obs.5 *ed *(*s*,*α*) + *ed *(*α*,*t*), and *ed *(*s*,*t*) ≤ Lem.8 *ed *(*s*^*a*^, *t*^*a*^) + *ed *(*s*^*b*^, *t*^*b*^) ≤ Obs.5 *ed *(*s*^*a*^, *t*^*a*^) + *ed *(*s*^*b*^, *α*) + *ed *(*α*,*t*^*b*^), therefore

$$\;\begin{array}{l} \text{ed}(s,t)\leq \text{min}\left\{\begin{array}{l} \text{ed}(s,\alpha )\;+\;\text{ed}(\alpha ,t),\\ \text{ed}(s^{a},t^{a})\;+\;\text{ed}(s^{b},\alpha )\;+\;\text{ed}(\alpha ,t^{b}) \end{array}\;\left|\;\begin{array}{l} (s^{a},s^{b})\in P(s),\\ (t^{a},t^{b})\in P(t),\\ \alpha \in \Sigma \end{array}\right)\;\right\}. \end{array}$$

Thus, to prove the correctness of Equation 7, it remains to show that

$$\;\begin{array}{l} \text{ed}(s,t)\;\geq \;\text{min}\left\{\;\begin{array}{l} \text{ed}(s,\alpha )+\text{ed}(\alpha ,t),\\ \text{ed}(s^{a},t^{a})+\text{ed}(s^{b},\alpha )+\text{ed}(\alpha ,t^{b}) \end{array}\;\left|\;\begin{array}{l} (s^{a},s^{b})\in P(s),\\ (t^{a},t^{b})\in P(t),\\ \alpha \in \Sigma \end{array}\right)\;\right\}. \end{array}$$

From Lemma 10, there is a simple script $ES=\langle s=u^{0},u^{1},\ldots ,u^{r}=t\rangle$ from *s* to *t*, and in particular, there is a string *u*^*i*^ along ES such that the partial script ${ES}^{0,i}$ is non-generating, and the partial script ${ES}^{i,r}$ is non-reducing. Recall that $\text{ed}(s,t)=\text{cost}(ES)=\text{cost}({ES}^{0,i})+\text{cost}({ES}^{i,r})\overset{\text{Obs.6}}{=}\text{ed}(s,u^{i})+\text{ed}(u^{i},t)$.

If *u*^*i *^= *β* for some letter *β*, then *ed *(*s*,*t*) = *ed *(*s*,*β*) + *ed *(*β*,*t*) ≥ min {*ed *(*s*,*α*) + *ed *(*α*,*t*) | *α* ∈ *Σ*}. Otherwise, *u*^*i*^ contains at least two letters. In this case, we can write *u*^*i*^ = *wβ*, where *β* is the last letter in *u*^*i*^ and *w* is the nonempty prefix of *u*^*i*^ containing all letters except for the last one. From Lemma 12, *ed *(*u*^*i*^,*t*) = *ed *(*w β*, *t*) = min {*ed *(*w*, *t*^*a*^) + *ed *(*β*, *t*^*b*^) | (*t*^*a*^, *t*^*b*^) ∈ *P *(*t*)}. Symmetrically, it is possible to show that *ed *(*s*, *u*^*i*^) = min {*ed *(*s*^*a*^, *w*) + *ed *(*s*^*b*^, *β*) | (*s*^*a*^, *s*^*b*^) ∈ *P *(*s*)}, and so

$$\;\begin{array}{ll} \text{ed}(s,t)\; & =\;\text{ed}(s,u^{i})\;+\;\text{ed}(u^{i},t)\\ & =\;\text{min}\left\{\text{ed}(s^{a},w)\;+\;\text{ed}(s^{b},\beta )\;|\;(s^{a},s^{b})\in P(s)\right\}\;+\;\\ & \;\text{min}\left\{\text{ed}(w,t^{a})\;+\;\text{ed}(\beta ,t^{b})\;|\;(t^{a},t^{b})\in P(t)\right\}\\ & \overset{\text{Obs.5}}{\geq }\;\text{min}\left\{\text{ed}(s^{a},t^{a})\;+\;\text{ed}(s^{b},\beta )\;+\;\text{ed}(\beta ,t^{b})|(s^{a},s^{b})\;\in \;P(s),\right)\\ & \;\left(\left(t^{a},t^{b})\in P(t\right)\right\}\\ & \geq \;\text{min}\left\{\;\begin{array}{l} \text{ed}(s,\alpha )\;+\;\text{ed}(\alpha ,t),\\ \text{ed}(s^{a},t^{a})\;+\;\text{ed}(s^{b},\alpha )\;+\;\text{ed}(\alpha ,t^{b}) \end{array}\;\left|\;\begin{array}{l} (s^{a},s^{b})\in P(s),\\ (t^{a},t^{b})\in P(t),\\ \alpha \in \Sigma \end{array}\right)\;\right\}, \end{array}$$

concluding the proof of Equation 7.

We next continue to develop the recursive computation, considering the simpler cases where one of the input strings is either empty or contains a single letter, and the other string contains at least two letters. Let ES be a simple script from *ε* to *t* whose length is *r*. ES must start with an insertion of some letter *α*, and from Observation 6, the remainder of the script ${ES}^{1,r}$ is an optimal script from *α* to *t*, implying the correctness of Equation 1. The correctness of Equation 4 is shown symmetrically.

Now, consider the computation of *ed *(*α*,*t*), as expressed in the last term of Equation 2. From Lemma 11, a simple script from *α* to *t* is non-reducing, and so the first operation in such a script is either the mutation of *α*, or some generating operation. If there is such a script in which the first operation is generating, then *ed *(*α*,*t*) = *ed *^′ ^(*α*,*t*) = *mut *(*α*,*α*) + *ed *^′ ^(*α*,*t*) ≥ min {*mut *(*α*,*β*) + *ed *^′ ^(*β*,*t*) | *β* ∈ *Σ*}. Else, there is a simple script from *α* to *t* in which the first operation is the mutation of *α* into some letter *β*. Due to Lemma 9, the following operation must be a generating operation, and so the reminder of the script is an optimal script from *β* to *t* in which the first operation is generating, implying again that *ed *(*α*,*t*) ≥ min {*mut *(*α*,*β*) + *ed *^′ ^(*β*,*t*) | *β* ∈ *Σ*}. The other direction of the inequality is shown similarly as done above for Equation 7, concluding the correctness proof of Equation 2.

We now address the correctness of Equation 3. Consider the minimum cost of a script from *α* to *t* which starts with a generating operation. Let ES be such a script, and let *r* denote its length.

For the case where the first operation in ES is an insertion of some letter *γ* after *α*, from Observation 6 we get that ${ES}^{1,r}$ is a non-reducing optimal script from *u*^1^ = *αγ* to *t*, and therefore in this case

$$\;\begin{array}{ll} ed^{\prime }(\alpha ,t) & =\;\text{cost}(ES)=\text{ins}(\gamma )+\text{ed}(\alpha \gamma ,t)\\ & \overset{\text{Lem.12}}{=}\;\text{min}\;\left\{\text{ins}(\gamma )+\text{ed}(\alpha ,t^{a})\right)\\ & \;\left(+\text{ed}(\gamma ,t^{b})\;|\;(t^{a},t^{b})\in P(t)\right\}\\ & \overset{\text{Obs.5}}{\geq }\;\text{min}\left\{\;,\text{ed}(\alpha ,t^{a})+\text{ed}(\varepsilon ,t^{b})|\;(t^{a},t^{b})\in P(t)\right\}. \end{array}$$

The cases where the first operation in ES is the insertion of some letter before *α*, or the duplication of *α*, are solved similarly and imply that

$$\;\begin{array}{l} ed^{\prime }(\alpha ,t)\;\geq \;\text{min}\;\left\{\;\left(\begin{array}{l} \text{ed}(\alpha ,t^{a})\;+\;\text{ed}(\varepsilon ,t^{b}),\\ \text{ed}(\varepsilon ,t^{a})\;+\;\text{ed}(\alpha ,t^{b}),\\ \text{dup}(\alpha )\;+\;\text{ed}(\alpha ,t^{a})\;+\;\text{ed}(\alpha ,t^{b}) \end{array}\right|(t^{a},t^{b})\in P(t)\;\right\} \end{array}$$

The other direction of the inequality is shown similarly as shown for Equation 7, concluding the proof for Equation 3. The correctness of Equations 5 and 6 is shown symmetrically.

### Correctness of Algorithm 2

We next show that when the precondition of COMPUTE-MATRIX holds with respect to its input region *I*_*i*, *k *_× *I*_*j*, *l*_, executing the procedure derives its postcondition, i.e. the procedure computes correctly all entries in the input region within *EDT*^*α *^and *ED*.

The base case of COMPUTE-MATRIX occurs when *k* = *i* + 1 and *l* = *j* + 1. In this case, *I*_*i*, *k *_= *i* and *I*_*j*, *l *_= *j*, and from the precondition we get that *EDT*^*α *^[ *i*,*j*] = *ED *[ *i*, *I*_1,*j*_] ⊗ *T*^*α *^[ *I*_1,*j*_,*j*] = Eq.13 *edt *^*α *^(*s*_0,*i*_,*t*_0,*j*_), and *ED *[ *i*,*j*] = min {*tr *(*S*^*α*^)[ *i*,*I*_1,*i*_] ⊗ *EDT*^*α *^[ *I*_1,*i*_,*j*] | *α* ∈ *Σ*}. After running line 2 of the procedure, we have from Equation 12 that *ED *[ *i*,*j*] = *ed *(*s*_0,*i*_,*t*_0,*j*_). Thus, all entries of the form *EDT*^*α *^[ *i*,*j*] and the entry *ED *[ *i*,*j*] are correctly computed, and the postcondition holds.

Else, either *k* > *i* + 1 or *l* > *j* + 1. In the case where *l* - *j* ≥ *k* - *i* (lines 5-8), the algorithm partitions vertically the region to be computed into two parts of approximately equal sizes. Let $h=\left\lceil \frac{j+l}{2}\right\rceil$ be the value computed in line 5 of the procedure. Note that from Item 1 of Observation 1, the fact that *EDT*^*α *^[ *I*_*i*,*k*_, *I*_*j*,*l*_] = *ED *[ *I*_*i*,*k*_, *I*_1,*j*_] ⊗ *T*^*α *^[ *I*_1,*j*_,*I*_*j*,*l*_] implies that *EDT*^*α *^[ *I*_*i*,*k*_,*I*_*j*,*h*_] = *ED *[ *I*_*i*,*k*_,*I*_1,*j*_] ⊗ *T*^*α *^[ *I*_1,*j*_,*I*_*j*,*h*_], and similarly *ED *[ *I*_*i*,*k*_,*I*_*j*,*h*_] = min {*tr*(*S*^*α*^)[ *I*_*i*,*k*_,*I*_1,*i*_] ⊗ *EDT*^*α *^[ *I*_1,*i*_,*I*_*j*,*h*_] | *α* ∈ *Σ*}. Thus, all requirements of the precondition with respect to the region *I*_*i*,*k *_× *I*_*j*,*h*_ are met, and the procedure is called recursively in line 6 over this region. From the postcondition of the recursive call, upon arriving to line 7 all entries in the region *I*_*i*,*k *_× *I*_*j*,*h*_ in matrices *EDT*^*α*^ and *ED* contain the solutions for the corresponding sub-instances. In particular, it may be observed that at this point of the run, all requirements for the precondition to hold with respect to the region *I*_*i*,*k *_ × *I*_*h*,*l*_ are met, with the exception of the requirements regarding entries in the region *I*_*i*,*k*_ × *I*_*h*,*l*_ of matrices *EDT*^*α*^. Again, from the precondition and Observation 1, at this stage *EDT*^*α *^[ *I*_*i*,*k*_,*I*_*h*,*l*_] = *ED *[ *I*_*i*,*k*_,*I*_1,*j*_]⊗*T*^*α*^[ *I*_1,*j*_,*I*_*h*,*l*_] for every *α* ∈ *Σ*. From Item 3 of Observation 1, min{*EDT*^*α *^[*I*_*i*,*k*_,*I*_*h*,*l*_ ],*ED *[*I*_*i*,*k*_,*I*_*j*,*h*_ ]⊗ *T*^*α*^[*I*_*j*,*h*_, *I*_*h*,*l*_] } = min {*ED *[ *I*_*i*,*k*_,*I*_1,*j*_]⊗*T*^*α *^[ *I*_1,*j*_,*I*_*h*,*l*_],*ED *[ *I*_*i*,*k*_,*I*_*j*,*h*_] ⊗ *T*^*α *^[ *I*_*j*,*h*_,*I*_*h*,*l*_]} = *ED *[ *I*_*i*,*k*_,*I*_1,*h*_] ⊗ *T*^*α*^[ *I*_1,*h*_,*I*_*h*,*l*_], and therefore after executing line 7, the precondition holds with respect to the region *I*_*i*,*k *_× *I*_*h*,*l*_. After returning from the recursive call in line 8, all entries in the region *I*_*i*,*k *_× *I*_*j*,*l*_ are computed, and the postcondition of the procedure is met. The correctness of the computation conducted lines 10-13 in the case where *l* - *j* < *k* - *i* is shown similarly.

Note that the initial call to COMPUTE-MATRIX from line 5 of Algorithm 2 is applied over the complete region *I*_*i*,*k *_× *I*_*j*,*l *_= *I*_2,*n*+1_ × *I*_2,*n*+1_. It may be observed that after the initialization in lines 3 and 4 of Algorithm 2, the precondition of COMPUTE-MATRIX is met with respect to this region. Therefore, it follows from the postcondition that once the computation terminates all entries in matrices *EDT*^*α*^ and *ED* contain the solutions for the corresponding sub-instances. In particular, *ED *[ *n*,*n*] holds the solution *ed *(*s*_0,*n*_,*t*_0,*n*_) = *ed *(*s*,*t*), and the returned value in line 6 of Algorithm 2 is correct.

### Proofs to lemmas corresponding to the EDDC algorithm for discrete cost functions

**Proof of lemma 1:** Matrix multiplications computed along the run of Algorithm 2 occur in lines 7 and 12 of Procedure COMPUTE-MATRIX, and additional implicit such multiplications occur when the Inside-VMT algorithm is used in Stage 1 of the algorithm. Note that in such computations, all entries in the multiplied sub-matrices already contain the computed solutions for the corresponding sub-instances. In addition, matrix multiplications conducted by the Inside-VMT algorithm are applied only over sub-matrices $A[\;I_{i_{1},i_{2}},I_{j_{1},j_{2}}]$ such that *i*_2_ ≤ *j*_1_ (see [11]), and thus, *D*-discreteness in matrices computed in Stage 1 need to be shown only with respect to adjacent entries *A *[ *i*,*j*], *A *[ *i* - 1,*j*] such that *i* ≤ *j*. In what follows, let 0 < *i* ≤ *n* and 0 ≤ *j* ≤ *n* be two integers for *n* the length of *s* and *t*.

Consider first the pair of adjacent entries *ED *[ *i*,*j*] and *ED *[ *i* - 1,*j*], which already contain the corresponding sub-instance solutions *ed *(*s*_0,*i*_,*t*_0,*j*_) and *ed *(*s*_0,*i*-1_,*t*_0,*j*_), respectively. An edit script transforming *s*_0,*i*-1_ to *t*_0,*j*_ can be composed by first inserting the letter *s*_*i*-1_ at the end of *s*_0,*i*-1_ to obtain the string *s*_0,*i*_ at cost *ins *(*s*_*i*-1_), and then transforming *s*_0,*i*_ to *t*_0,*j*_ by applying an optimal script at cost *ed*(*s*_0,*i*_,*t*_0,*j*_). Therefore, *ed *(*s*_0,*i*-1_,*t*_0,*j*_) ≤ *ins *(*s*_*i*-1_) + *ed *(*s*_0,*i*_,*t*_0,*j*_). Also, an edit script transforming *s*_0,*i*_ to *t*_0,*j *_can be composed by first deleting last letter *s*_*i*-1_ from *s*_0,*i*_ at cost *del *(*s*_*i*-1_), and then transforming *s*_0,*i*-1_ to *t*_0,*j*_ at cost *ed *(*s*_0,*i*-1_,*t*_0,*j*_). Therefore, *ed *(*s*_0,*i*_,*t*_0,*j*_) ≤ *del *(*s*_*i*-1_) + *ed*(*s*_0,*i*-1_,*t*_0,*j*_). Thus, *a* ≤ - *del *(*s*_*i*-1_) ≤ *ed *(*s*_0,*i*-1_,*t*_0,*j*_) - *ed *(*s*_0,*i*_,*t*_0,*j*_) ≤ *ins *(*s*_*i*-1_) < *b*. Since all operation costs are integers, the cost of any edit script is an integer. Hence, after the adjacent entries *ED *[ *i* - 1,*j*] and *ED *[ *i*,*j*] are computed, *ED *[ *i* - 1,*j*] - *ED *[ *i*,*j*] = *ed *(*s*_0,*i*-1_,*t*_0,*j*_) - *ed *(*s*_0,*i*_,*t*_0,*j*_) is an integer within the interval *D* = *I*_*a*,*b*_. The *D*-discreteness proofs for computed sub-matrices in all matrices of the form *T*^′*α*^, *T*^*α*^, *T*^*ε*^, *S*^′*α*^, *S*^*α*^, *S*^*ε*^ (as well as for the transformed matrix *tr *(*S*^*α*^)) are obtained similarly.

For the matrix *EDT*^*α*^, note that there exists an integer *l*^∗^ such that $\text{ed}t^{\alpha }(s_{0,i-1},t_{0,j})\overset{\text{Eq.9}}{=}\text{ed}(s_{0,i-1},t_{0,l^{*}})+\text{ed}(\alpha ,t_{l^{*},j})$. In addition, in the same manner as above, for the same *l*^∗^ we get that $\text{ed}t^{\alpha }\left(s_{0,i},t_{0,j}\right)\overset{\text{Eq.9}}{\leq }\text{ed}(s_{0,i},t_{0,l^{*}})+\text{ed}(\alpha ,t_{l^{*},j})\leq \text{del}(s_{i-1})+\text{ed}(s_{0,i-1},t_{0,l^{*}})+\text{ed}(\alpha ,t_{l^{*},j})=\text{del}(s_{i-1})+\text{ed}t^{\alpha }(s_{0,i-1},t_{0,j})$.Similarly, it can be shown that *edt *^*α *^(*s*_0,*i*-1_, *t*_0,*j*_) ≤ *ins *(*s*_*i*-1_) + *edt *^*α *^(*s*_0,*i*_,*t*_0,*j*_), and so after the entries *EDT *^*α*^[ *i* - 1,*j*] and *EDT*^*α *^[ *i*,*j*] are computed, *EDT*^*α *^[ *i* - 1,*j*] - *EDT*^*α *^[ *i*,*j*] = *edt*^*α *^(*s*_0,*i*-1_, *t*_0,*j*_) - *edt *^*α *^(*s*_0,*i*_,*t*_0,*j*_) is an integer within *D*.

**Proof of lemma** 2**:** Consider a pair of adjacent entries *Z *[ * i*- 1,*j*],*Z *[ *i*,*j*] in *Z*. Let *r*_1_ and *r*_2_ be indices, such that *Z *[ *i* - 1,*j*] = *X *[ *i* - 1,*r*_1_] + *Y *[ *r*_1_,*j*] and *Z *[ *i*,*j*] = *X *[ *i*,*r*_2_] + *Y *[ *r*_2_,*j*]. Then:

$$\;\begin{array}{ll} Z[\;i-1,j]-Z[\;i,j] & =X[\;i-1,r_{1}]+Y[\;r_{1},j]-\left(X[\;i,r_{2}]+Y[\;r_{2},j]\right)\\ & \leq X[\;i-1,r_{2}]+Y[\;r_{2},j]-\left(X[\;i,r_{2}]+Y[\;r_{2},j]\right)\\ & =X[\;i-1,r_{2}]-X[\;i,r_{2}]<\mathrm{b.} \end{array}$$

Similarly, it can be shown that *Z *[ *i* - 1,*j*] - *Z *[ *i*,*j*] ≥ *a*. Since *X* and *Y* contain only integer entries, it follows that *Z* contains only integer entries, and thus *Z *[ *i* - 1,*j*] - *Z *[ *i*,*j*] is an integer within *D*.

**Proof of lemma** 3**:** Consider a pair of adjacent entries *Z *[ *i* - 1,*j*],*Z *[ *i*,*j*] in *Z*. Then:

$$\begin{array}{ll} Z[\;i-1,j]-Z[\;i,j] & =\text{min}\{X[\;i-1,j],Y[\;i-1,j]\}\\ & \;-\text{min}\{X[\;i,j],Y[\;i,j]\}\\ & <\text{min}\{X[\;i,j]+b,Y[\;i,j]+b\}\\ & \;-\text{min}\{X[\;i,j],Y[\;i,j]\}=\mathrm{b.} \end{array}$$

Similarly, it can be shown that *Z *[ *i* - 1,*j*] - *Z *[ *i*,*j*] ≥ *a*. Since *X* and *Y* contain only integer entries, it follows that *Z* contains only integer entries, and thus *Z *[ *i* - 1,*j*] - *Z *[ *i*,*j*] is an integer within *D*.

**Proof of lemma** 4**:** Since both *x* and *y* are *D*-discrete, for every 0 < *i* < *q*, *x*_*i*_ < *x*_0_ + *ib *and *y*_0_ + *ia *≤ *y*_*i*_. Hence, *x*_*i*_ < *x*_0_ + *ib *= *x*_0_ + *ib* + (*y*_0_ - *y*_0_ + *ia *- *ia*) = (*y*_0_ + *ia*) + *i *(*b* - *a*) - (*y*_0_ - *x*_0_) < *y*_*i*_ + *q*|*D*| - (*y*_0_ - *x*_0_). Therefore, when *y*_0_ - *x*_0_ ≥ *q*|*D*|, *x*_*i *_< *y*_*i*_ for every 0 ≤ *i* < *q*.

### Proofs to lemmas corresponding to the run-length encoding based EDDC algorithm

**Proof of lemma** 5**:** We show that *ed *(*α*,*β w*) ≥ *ed *(*α*,*w*) + *dup *(*β*), where the other inequalities are proven similarly.

Let *r* be the length of a simple script from *α* to *β w*. Observe that *r* ≥ 1, since by definition *β w* ≠ *α*. When *r* = 1, the single operation applied over *α* must be a generating operation (since *w* ≠ *ε*). As discussed in Section “Additional acceleration using run-length encoding”, we may assume this operation is the duplication of *α*, and so *β* = *w* = *α*. In this case, *ed *(*α*,*β w*) = *dup *(*α*) = *ed *(*s*,*w*) + *dup *(*β*).

When *r* > 1, assume by induction the lemma holds for every instance such that the length of a simple script from the source to the target string is less than *r*. A simple script from *α* to *β w* is non-reducing (as shown in Section “Correctness of the recursive computation”). If there is such a script in which the first operation is a mutation, then this operation mutates *α* into some letter *γ* ≠ *α*, and the remainder of the script is a simple script of length *r* - 1 from *γ *to *β w*. In this case, the inductive assumption implies that *ed *(*α*,*β w*) = *mut *(*α*,*γ*) + *ed *(*γ*,*β w*) ≥ *mut *(*α*,*γ*) + *ed *(*γ*,*w*) + *dup *(*β*) ≥ Obs.5 *ed *(*α*,*w*) + *dup *(*β*). Otherwise, there is a simple script from *α* to *β w* which starts with a generating operation. Again, we may assume this generating operation is the duplication of *α*. As shown in Section “Correctness of the recursive computation”, this implies that *ed *(*α*,*β w*) = *dup *(*α*) + *ed *(*α*,*t*^*a*^) + *ed *(*α*,*t*^*b*^) for some partition (*t*^*a*^,*t*^*b*^) ∈ *P *(*βw*), where the lengths of simple scripts from *α* to *t*^*a*^ and to *t*^*b*^ are strictly shorter than *r*. If *t*^*a*^ = *β* and *t*^*b*^ = *w*, then *ed *(*α*,*β w*) = *dup *(*α*)+  *ed *(*α*,*β*) + *ed *(*α*,*w*) = *dup *(*α*) + *mut *(*α*,*β*) + *ed *(*α*,*w*) ≥ Const.1 *ed *(*α*,*w*) + *dup *(*β*). Otherwise, *t*^*a*^ is of the form *β u* and *w* = *ut*^*b*^ for some string *u* ≠ *ε*. From the inductive assumption, *ed *(*α*,*β w*) = *dup *(*α*) + *ed *(*α*,*β u*) + *ed *(*α*,*t*^*b*^) ≥ *dup *(*α*) + *ed *(*α*,*u*) + *dup *(*β*) + *ed *(*α*,*t*^*b*^) ≥ Lem.8 *dup *(*α*) + *ed *(*α α*,*w*) + *dup *(*β*) ≥ Obs.5 *ed *(*α*,*w*) + *dup *(*β*).

**Proof of lemma** 6**:** Note that if *w* = *ε* or *w* = *β* for some *β* ∈ *Σ*, then $\tilde{w}=w$, *dupcost *(*w*) = *contcost *(*w*) = 0, and the lemma holds in a straightforward manner. Otherwise, *w* is of length at least 2, and we prove the lemma by induction over the length *r* of a simple script between *α* and *w*. Assume by induction the lemma holds for every pair of input strings such that the length of a simple script from the source to the target string is less than *r*. We show that $\text{ed}(\alpha ,w)=\text{ed}(\alpha ,\tilde{w})+\text{dupcost}(w)$, where the proof that $\text{ed}(w,\alpha )=\text{contcost}(w)+\text{ed}(\tilde{w},\alpha )$ is symmetric.

Observe that $\text{ed}(\alpha ,w)\overset{\text{Obs.5}}{\leq }\text{ed}(\alpha ,\tilde{w})+\text{ed}(\tilde{w},w)\leq \text{ed}(\alpha ,\tilde{w})+\text{dupcost}(w)$, and therefore it remains to show that $\text{ed}(\alpha ,w)\geq \text{ed}(\alpha ,\tilde{w})+\text{dupcost}(w)$. As discussed in the proof of Lemma 5, there is a simple script from *α * to *w* which either starts with a mutation of *α* or its duplication. If the first operation in such a script is the mutation of *α* to some letter *β *∈ *Σ*, then the remainder of the script is a simple script from *β* to *w* of length *r* - 1, and from the inductive assumption $\text{ed}(\alpha ,w)=\text{mut}(\alpha ,\beta )+\text{ed}(\beta ,w)=\text{mut}(\alpha ,\beta )+\text{ed}(\beta ,\tilde{w})\;+\;\text{dupcost}(w)\overset{\text{Obs.5}}{\geq }\text{ed}(\alpha ,\tilde{w})\;+\;\text{dupcost}(w)$. Otherwise, the first operation is the duplication of *α*, and there is some partition (*w*^*a*^,*w*^*b*^) ∈ *P *(*w*) such that *ed *(*α*,*w*) = *dup *(*α*) + *ed *(*α*,*w*^*a*^) + *ed *(*α*,*w*^*b*^) and the sum of lengths of shortest optimal scripts from *α* to *w*^*a*^ and to *w*^*b*^ is *r* - 1. From the inductive assumption, $\text{ed}(\alpha ,w^{a})=\text{ed}(\alpha ,{\tilde{w}}^{a})+\text{dupcost}(w^{a})$ and $\text{ed}\left(\alpha ,w^{b}\right)=\text{ed}\left(\alpha ,{\tilde{w}}^{b}\right)+\text{dupcost}(w^{b})$. If (*w*^*a*^,*w*^*b*^) ∈ *R *(*w*), then $\tilde{w}={\tilde{w}}^{a}{\tilde{w}}^{b}$ and *dupcost *(*w*) = Eq.18 *dupcost *(*w*^*a*^) + *dupcost *(*w*^*b*^). In this case, $\text{ed}(\alpha ,w)=\text{dup}(\alpha )+\left(\text{ed}(\alpha ,{\tilde{w}}^{a})+\text{dupcost}(w^{a})\right)+\left(\text{ed}(\alpha ,{\tilde{w}}^{b})+\text{dupcost}(w^{b})\right)\overset{\text{Lem.8}}{\geq }\text{dup}(\alpha )+\text{ed}(\alpha \alpha ,\tilde{w})+\text{dupcost}(w)\overset{\text{Obs.5}}{\geq }\text{ed}(\alpha ,\tilde{w})+\text{dupcost}(w)$. Else, (*w*^*a*^,*w*^*b*^) ∉ *R *(*w*), and so there is some letter *β *∈ *Σ* such that *w*^*a*^ ends with *β* and *w*^*b*^ starts with *β*. In this case, there are some strings *u*^*a*^,*u*^*b*^ and integers *p*,*q* > 0, such that *w*^*a*^ = *u*^*a *^*β*^*p*^, *w*^*b *^= *β*^*q *^*u*^*b*^, *u*^*a*^ does not end with *β*, and *u*^*b*^ does not start with *β*. Moreover, ${\tilde{w}}^{a}={ũ}^{a}\beta$, ${\tilde{w}}^{b}=\beta {ũ}^{b}$, and $\tilde{w}={ũ}^{a}\beta {ũ}^{b}={\tilde{w}}^{a}{ũ}^{b}$. Note that $\text{ed}(\alpha ,{\tilde{w}}^{b})=\text{ed}(\alpha ,\beta {ũ}^{b})\overset{\text{Lem.5}}{\geq }\text{ed}(\alpha ,{ũ}^{b})+\text{dup}(\beta )$, and therefore $\text{ed}(\alpha ,w)=\text{dup}(\alpha )+\text{ed}(\alpha ,w^{a})+\text{ed}(\alpha ,w^{b})=\text{dup}(\alpha )+\left(\text{ed}(\alpha ,{\tilde{w}}^{a})+\text{dupcost}(w^{a})\right)+\left(\text{ed}(\alpha ,{\tilde{w}}^{b})+\text{dupcost}(w^{b})\right)\geq \text{dup}(\alpha )+\text{ed}(\alpha ,{\tilde{w}}^{a})+\text{ed}(\alpha ,{ũ}^{b})$$+\;\text{dup}(\beta )+\text{dupcost}(w^{a})+\text{dupcost}(w^{b})\overset{\text{Eq.18}}{=}\text{dup}(\alpha )+\text{ed}(\alpha ,{\tilde{w}}^{a})+\text{ed}(\alpha ,{ũ}^{b})+\text{dupcost}(w)\overset{\text{Lem.8}}{\geq }\text{dup}(\alpha )+\text{ed}(\alpha \alpha ,\tilde{w})+\text{dupcost}(w)\overset{\text{Obs.5}}{\geq }\text{ed}(\alpha ,\tilde{w})+\text{dupcost}(w)$.

## Competing interests

The authors declare that they have no competing interests.

## Authors’ contributions

All authors developed the algorithms, drafted the manuscript, read and approved the final manuscript.
